# Supplementary material for: Disruption of the tumour-associated EMP3 enhances erythroid proliferation and causes the MAM-negative phenotype
Source: Nat Commun. 2020 Jul 16;11:3569. doi: 10.1038/s41467-020-17060-4 (PMC7366909; doi:10.1038/s41467-020-17060-4)
Supplement: Supplementary file 1 — Supplementary Information [file 41467_2020_17060_MOESM1_ESM.pdf]

Supplementary Information

Disruption of the tumour-associated *EMP3* enhances erythroid proliferation and causes the MAM-negative phenotype

Nicole Thornton[1†], Vanja Karamatic Crew[1†], Louise Tilley[1†], Carole A. Green[2], Chwen Ling Tay[1], Rebecca E. Griffiths[2], Belinda K. Singleton[2], Frances Spring[2], Piers Walser[1], Abdul Ghani Alattar[3], Benjamin Jones[1], Rosalind Laundy[1], Jill R. Storry[3,4], Mattias Möller[3], Lorna Wall[5], Richard Charlewood[5], Connie M. Westhoff[6], Christine Lomas-Francis[6], Vered Yahalom[7], Ute Feick[8], Axel Seltsam[9], Beate Mayer[10], Martin L. Olsson[3,4‡], David J. Anstee[2‡]*

[1]International Blood Group Reference Laboratory, NHS Blood and Transplant, Bristol, UK
[2]Bristol Institute for Transfusion Sciences, NHS Blood and Transplant and NIHR Blood and Transplant Unit in Red Cell Products, University of Bristol, Bristol, UK
[3]Division of Hematology and Transfusion Medicine, Department of Laboratory Medicine, Lund University, Lund, Sweden
[4]Department of Clinical Immunology and Transfusion Medicine, Office of Medical Services, Lund, Sweden
[5]Reference Laboratory, New Zealand Blood Service, Auckland, New Zealand
[6]New York Blood Centre, New York, USA
[7]Magen David Adom, National Blood Services, Ramat Gan, Israel
[8]Deutsches Rotes Kreuz, Blood Donor Service, Institute Bad Kreuznach, Germany
[9]German Red Cross Blood Service NSTOB, Institute Springe, Germany
[10]Institute of Transfusion Medicine, Charité-Universitätsmedizin Berlin, corporate member of Freie Universität Berlin, Humboldt-Universität zu Berlin, and Berlin Institute of Health, Germany
[†]shared first authors, [‡]shared senior authors

Contents

**Supplementary Table 1**	List of samples
**Supplementary Table 2**	Serology of MAM-negative samples
**Supplementary Table 3**	In^b and INFI (CD44) typing
**Supplementary Table 4**	MAIEA assay
**Supplementary Table 5**	Summary of detected *EMP3* inactivating mutations
**Supplementary Table 6**	Nonsense *EMP3* variants in gnomAD database
**Supplementary Table 7**	*EMP3* alleles in 1000 Genomes Project dataset
**Supplementary Table 8**	Relative EMP3 expression in BEL-A2 (proteomics)
**Supplementary Table 9**	Serological testing of anti-MAM and anti-EMP3
**Supplementary Table 10**	Inhibition of anti-EMP3 binding to MAM
**Supplementary Table 11**	EMP3 up- and down-regulation in diverse cancers
**Supplementary Table 12**	List of *EMP3* and *CD44H* primers
**Supplementary Table 13**	Parameters for protein modelling
**Supplementary Figure 1**	CD44 expression by flow cytometry
**Supplementary Figure 2**	shRNA *EMP3* experiments
**Supplementary Figure 3**	*EMP3* KO experiments; staining with anti-EMP3
**Supplementary Figure 4**	EMP3 distribution in human tissues and cell lines
**Supplementary Figure 5**	MAM expression on platelets
**Supplementary Figure 6**	Additional cell culture experiments
**Supplementary Figure 7**	CD44 and MAM immunoblotting and co-IP
**Supplementary Figure 8**	MAM and CD44 expression in cell culture
**Supplementary Figure 9**	*CD44* KO experiments
**Supplementary Figure 10**	Flow cytometry gating strategy
**Supplementary Figure 11**	Uncropped original immunoblotting images

Supplementary References
Supplementary Proteomics Data File 1

Supplementary Table 1. MAM-negative patient samples (coded P1-P10) and P9's family samples (coded P9-mother, P9-father, P9-sibling) included in the study.

**Sample**	**Year of Discovery**	**Referred from**<br><br>***Ethnicity***	**Clinical Information**	**Reference**
P1	1993 (Original)	IBGRL collection<br><br>*Partial Irish and Cherokee descent*	First two children not affected. Antibody detected during 3rd pregnancy, no clinical evidence of HDFN	Anderson *et al*.[1]<br>Montgomery *et al*.[2]
P2	2000	IBGRL collection<br><br>*Arabic*	3rd pregnancy, severe HDFN.<br>At 29 weeks gestation, during IUT, emergency caesarean section required. Baby died at 6 months old due to complications of premature birth (posttraumatic pulmonary insufficiency)	Montgomery *et al*.[2]
P3 (sibling of P2)	2000	New York, USA<br><br>*Arabic*	One successful pregnancy	Montgomery *et al*.[2]
P4	2001	Berlin, Germany<br><br>*Not known*	No data	None
P5	2003	Bad Kreuznach, Germany<br><br>*Turkish*	No data	None
P6	2012	New York, USA<br><br>*Yemeni*	Patient was G2, P1<br>Baby born full-term at 38 weeks.<br>Laboratory values at birth: Hb 21 g/dL (range: 13.5-19.5 g/dL), platelet count 132,000/uL (range: 150,000-450,000/uL), total bilirubin 7.1 mg/dL (range: 6-8 mg/dL).<br>No indication for RBCs/platelets transfusion or UV phototherapy.<br>Newborn discharged from hospital on day 3 of life without evidence of any clinical symptoms.	Burgos *et al*.[3]
P7	2014	New York, USA<br><br>*Caucasian from Virginia*	Female patient born in 1960 with history of pregnancy. Diagnosis included sepsis, colitis, UTI, small bowel resection.	None
P8	2015	Berlin, Germany<br><br>*Kurdish Turk*	Antibody detected during 2nd pregnancy, no clinical evidence of HDFN, newborn's RBCs DAT negative.	None
P9	2015	Auckland, New Zealand<br><br>*Caucasian*	Severe HDFN, required IUT. Placental abruption and IU death at 28 weeks.	None
P9-mother (mother of P9)	2015	Auckland, New Zealand	Not applicable	None
P9-father (father of P9)	2015	Auckland, New Zealand	Not applicable	None
P9-sibling (sibling of P9)	2015	Auckland, New Zealand	Not applicable	None
P10	2015 (first detected in 1994)	Sweden<br><br>*Middle Eastern*	Two children, not affected	None

Supplementary Table 2. Serological characterisation of MAM.

Red Cell Antigen Molecules	CD Number	Blood Group System		
Glycophorin A	CD235a	MNS	●	Δ
Glycophorin B	CD235b	MNS	●	Δ
α-1,4-Galactosyltransferase	CD77	P1PK	nt	Δ
RhD	CD240D	RH	●	Δ
RhCcEe	CD240CE	RH	●	Δ
Basal Cell Adhesion Molecule	CD239	LU	●	Δ
Kell Metallo-Endopeptidase	CD238	KEL	●	Δ
Fucosyltransferase 3	CD174	LE	●	Δ
Atypical Chemokine Receptor 1	CD234	FY	●	Δ
Solute Carrier Family 14 Member 1		JK	●	Δ
Solute Carrier Family 4 Member 1	CD233	DI	●	Δ
Acetylcholinesterase		YT	●	Δ
CD99	CD99	XG	●	nt
Erythroblast Membrane Associated Protein		SC	●	Δ
ADP-Ribosyltransferase 4	CD297	DO	●	Δ
Aquaporin 1		CO	●	Δ
Intercellular Adhesion Molecule 4	CD242	LW	●	Δ
Complement C4B		CH/RG	●	Δ
Complement C4A		CH/RG	●	Δ
Fucosyltransferase 1		H	●	Δ
Kx Protein		XK	●	Δ
Glycophorin C	CD236	GE	●	Δ
CD55	CD55	CROM	●	Δ
Complement C3b/C4b Receptor 1	CD35	KN	●	Δ
CD44	CD44	IN	**w**	Δ
Basigin	CD147	OK	●	Δ
CD151	CD151	RAPH	●	Δ
Semaphorin 7A	CD108	JMH	●	Δ
Glucosaminyl (N-Acetyl) Transferase 2		I	●	Δ
β-1,3-N-Acetylgalactosaminyltransferase 1		GLOB	●	Δ
Aquaporin 3		GIL	●	nt
Rh Associated Glycoprotein	CD241	RHAG	●	Δ
ATP Binding Cassette Subfamily G Member 2	CD338	JR	●	Δ
ATP Binding Cassette Subfamily B Member 6		LAN	●	Δ
Small Intergral Membrane Protein 1		VEL	●	Δ
CD59	CD59	CD59	nt	Δ
Solute Carrier Family 29 Member 1		AUG	●	Δ
Prion Protein	CD230	KANNO	●	Δ
β-1,4-N-Acetyl-Galactosaminyltransferase 2		SID	nt	Δ
		**High Prevalence RBC Antigens (no system)**		
unknown		Cs^{a}	●	Δ
unknown		Er^{a}	●	Δ
unknown		Er3	nt	Δ
unknown		LKE	nt	Δ
unknown		Emm	●	Δ
unknown		AnWj	●	Δ
unknown		PEL	●	Δ
unknown		ABTI	●	Δ

Key: anti-MAM found to be positive (Δ) with RBCs lacking high prevalence antigens of this molecule; MAM-negative cells found to be positive (●) or weak (**w**) with antibody(ies) detecting presence of this molecule. nt, not tested.

Supplementary Table 3. Serological typing of red blood cells (RBCs) from five MAM-negative patients (P2, P4, P8, P9 and P10) and MAM-positive control cells, with anti-In^b and anti-INFI (antibodies reacting with antigens of Indian blood group system, carried on CD44).

RBC samples	Antibodies	
	Anti-In^b	Anti-INFI
**P2**	0	1+
**P4**	1+	w
**P8**	(+)	nt
**P9**	2+	nt
**P10**	1+	nt
**Control MAM+**	4+	4+

nt, not tested

Supplementary Table 4. MAIEA results with anti-MAM (P1).

Antibody Clone	Protein Specificity	Absorbance Ratio (>2.0 = positive)
BRIC222*****	CD44 (epitope 1)	16.5
BRIC241	CD44 (epitope 1)	21.0
BRIC205	CD44 (epitope 2)	14.3
BRIC214	CD44 (epitope 2)	13.4
BRIC219	CD44 (epitope 2)	14.8
BRIC223	CD44 (epitope 2)	20.4
BRIC225	CD44 (epitope 2)	14.9
BRIC235*****	CD44 (epitope 2)	21.4
KZ1*****ᵼ	CD44 (epitope 3)	35.3
BRIC224	Lu (domain 1)	1.0
BRIC221	Lu (domain 4)	1.1
BRIC18	Kell (K18 region)	1.0
BRIC203	Kell ($Kp^{b/c}$)	1.2
HIM6	CD147 (domain 1 epitope	1.0
UM-8D6	CD147 (domain 1 epitope	1.1
MEM6/6	CD147 (domain 2)	1.0
LA1818	RhAG	1.5
AE-1	AChE	1.0
BRIC230	DAF (SCR-1)	1.2
BRIC110	DAF (SCR-2)	1.1
BRIC5	LFA-3	1.0
BRIC229	CD59 (epitope 2)	1.0
BRIC6	Band 3	1.0
BIRMA84b	Band 3 (Wr^b)	1.2
BRIC125	CD47	0.9
MIMA123	Dombrock	1.0

*****Also tested with P4 anti-MAM and found positive; ᵼ also tested with P2 and P5 anti-MAM and found positive.

Supplementary Table 5. Summary of *EMP3* inactivating mutations identified in all MAM negative samples tested (P1-P10) and family members of P9 (P9-mother, P9-father, P9-sibling).

Sample	*EMP3** mutation	Methods used	Notes
P1	Homozygous full gene deletion	PCR, Sanger sequencing	No amplification of *EMP3* exons 2 to 5. Amplification of 700bp product as seen in P9 suggests presence of same/similar deleted allele. No wild-type allele present, but compound heterozygosity for two different deletions remains possible. Sanger sequencing shows breakpoints to be slightly different from P9, with a deletion of 8518bp (c.1-3513_492+1379del).
P2	Homozygous c.[123C>G; 373A>G] (p.Tyr41Ter)	Exome sequencing, PCR, Sanger sequencing	Premature stop codon, predicting no expression of functional EMP3. Additional variant c.373A>G after predicted stop codon will not be expressed on truncated protein.<br>The gnomAD database shows *EMP3* c.123G allele freq. at 43/251,000 (0.00017; predicted homozygote freq. $2.9x10^{-8}$).
P3<br>(sibling of P2)	Homozygous c.[123C>G; 373A>G] (p.Tyr41Ter)	PCR, Sanger sequencing	See notes for P2.
P4	Homozygous exon 5 deletion	Exome sequencing, PCR, Sanger sequencing	No coverage of *EMP3* exon 5 on exome sequence. No apparent loss of read depth in remaining areas of *EMP3*, supporting homozygosity for exonic deletion (rather than hemizygosity with whole gene deletion). No amplification with exon 5 primers (exon 2, 3, 4 amplify with no mutations). Amplification with *EMP*3 Ex4F and *TMEM143*R shows band of reduced size- from expected 2807bp to around 2000bp, suggesting deletion of ~800bp encompassing *EMP3* exon 5. Sanger sequencing shows deletion of 822bp, encompassing all of exon 5 (c.323-231_*421del).
P5	Homozygous exon 4 deletion	Exome sequencing, PCR, Sanger sequencing	No coverage of *EMP3* exon 4 on exome sequence. No apparent loss of read depth in remaining areas of *EMP3*, supporting homozygosity for exonic deletion (rather than hemizygosity with whole gene deletion). No amplification with exon 4 primers (exon 2, 3, 5 amplify with no mutations). Amplification with *EMP3* Ex3F and Ex5R shows product of reduced size- from expected 3312bp to around 2600bp, suggesting deletion of ~700bp encompassing *EMP3* exon 4. Sanger sequencing shows deletion of 745bp, encompassing all of exon 4 (c.182-186_322+418del).
P6	Homozygous c.[123C>G; 373A>G] (p.Tyr41Ter)	PCR, Sanger sequencing	See notes for P2.
P7	Homozygous full gene deletion	PCR, Sanger sequencing	See notes for P1.
P8	Homozygous exon 4 deletion	Exome sequencing, PCR, Sanger sequencing	See notes for P5.
P9	Homozygous full gene deletion	Exome sequencing, PCR, Sanger sequencing	No *EMP3* coverage on exome sequencing. No amplification of *EMP3* exons 2 to 5. Primers spanning a predicted ~9000bp region encompassing *EMP*3 amplify a ~700bp band (not seen in controls), suggesting a deletion of >8000bp. Sanger sequencing shows deletion of 8519bp, spanning complete *EMP3* gene (c.1-3532_*1361del). Data from parents (P9-mother and P9-father) suggests P9 may be a compound heterozygote for two similar, but genetically distinct, whole gene deletions (see below).
P9-mother	Presumed heterozygous for full gene deletion, with wild-type allele	Exome sequencing, PCR, Sanger sequencing	*EMP3* coverage on exome sequencing reduced by approx 50%. Presence of (at least) one copy of *EMP3* gene confirmed by PCR. Amplification of 700bp band as seen in P9 shows presence of deleted allele (presumed heterozygous with wild-type *EMP3* allele). Sanger sequencing confirms heterozygosity for P9 deletion (c.1-3532_*1361del).
P9-father	Presumed heterozygous for full gene deletion, with wild-type allele	Exome sequencing, PCR, Sanger sequencing	*EMP3* coverage on exome sequencing reduced by approx 50%. Presence of (at least) one copy of *EMP3* gene confirmed by PCR. No 700bp band as seen in P9 apparent- suggesting a larger deletion encompassing these primer sites. Breakpoints have not been identified by Sanger sequencing.
P9-sibling	Presumed heterozygous for full gene deletion, with wild-type allele	Exome sequencing, PCR, Sanger sequencing	*EMP3* coverage on exome sequencing reduced by approx 50%. Presence of (at least) one copy of *EMP3* gene confirmed by PCR. Amplification of 700bp band as seen in P9 shows presence of deleted allele- inherited from mother, presumably with wild-type allele from father. Sequencing confirms heterozygosity for P9 deletion (c.1-3532_*1361del).
P10	Homozygous c.[123C>G; 373A>G] (p.Tyr41Ter)	PCR, Sanger sequencing	See notes for P2.

**EMP3* reference sequence NM_001425.2, five exon transcript, coding exons 2-5.

Supplementary Table 6. Nonsense variants in *EMP3* found in the gnomAD exome dataset v2.1.1[4] (no loss-of-function homozygous variants recorded). NM_001425.2 was used as the reference sequence. The gnomAD database uses the ENST00000270221.6 transcript with coding exons 2-5 identical to NM_001425.2 but with a slightly longer non-coding exon 1.

Variant ID	Nucleotide change	Predicted amino acid change	gnomAD allele frequency (%)	
rs777112006	c.1A>G	p.Met1?	0.0014	(4 of 282,756)*
rs146324824	c.3G>C	p.Met1?	0.00040	(1 of 251,438)*
rs747282198	c.78+1G>A	Altered splicing	0.00080	(2 of 251,446)
rs747282198	c.78+1G>C	Altered splicing	0.00040	(1 of 251,446)
rs748935769	c.85_86delTG	p.Trp29AspfsTer27	0.00040	(1 of 249,144)
rs768347751	c.92_93delTC	p.Leu31ProfsTer25	0.00040	(1 of 249,668)
rs201392469	c.123C>G	p.Tyr41Ter	0.017	(43 of 251,000)**
rs1269751062	c.156G>A	p.Trp52Ter	0.00040	(1 of 251,012)
rs778509172	c.172delA	p.Ser58AlafsTer6	0.00040	(1 of 250,700)
rs1372215797	c.181+2T>C	Altered splicing	0.00040	(1 of 249,932)
rs761028282	c.182-2A>C	Altered splicing	0.00080	(2 of 251,258)
rs764214118	c.182-1G>A	Altered splicing	0.00080	(2 of 251,264)
rs1337327394	c.187_202del16	p.Leu63SerfsTer49	0.00040	(1 of 251,298)
rs758799081	c.217_218dupTC	p.Leu74ProfsTer44	0.00040	(1 of 251,424)
rs1394101002	c.247delC	p.Leu83CysfsTer34	0.00080	(2 of 251,452)
rs202222961	c.274C>T	p.Arg92Ter	0.00080	(2 of 251,238)
rs763027671	c.277C>T	p.Arg93Ter	0.0016	(4 of 251,250)
rs775496329	c.364delG	p.Ala122ProfsTer?	0.00041	(1 of 241,670)
N/A	c.390delG	p.Arg131GlufsTer?	0.0011	(3 of 274,744)
rs1471461966	c.432_453dupGGCCTTC CCCCTCGCCCTGGTC	p.Ser152GlyfsTer46	0.00042	(1 of 237,216)
rs763479095	c.437delT	p.Phe146SerfsTer?	0.00042	(1 of 236,416)
rs769824313	c.492A>G	p.Ter164TrpextTer23	0.00049	(1 of 204,384)

* Start codon lost. Next methionine is found 69bp downstream.

** Detected in multiple studies. Twenty-five of these alleles have been detected in Ashkenazi Jews (0.25% allele frequency in this group).

Supplementary Table 7. *EMP3* alleles found among the 2,504 individuals included in the 1000 Genomes Project[5]. NM_001425.2 was used as the reference sequence. Only one allele with a probable inactivating variant was found in this dataset. The impact on MAM antigen expression for other alleles is not known.

Allele count (total n=5,008)		Nucleotide change	Amino acid change	AFR		AMR		EAS		EUR		SAS	
4,917	(98.2%)			1,320	(99.8%)	678	(97.7%)	1,004	(99.6%)	959	(95.3%)	956	(97.8%)
77	(1.54%)	c.373A>G	p.Ile125Val	0		16	(2.31%)	0		46	(4.57%)	15	(1.53%)
5	(0.10%)	c.210G>A	p.Met70Ile	0		0		0		0		5	(0.51%)
3	(0.06%)	c.278G>A	p.Arg93Gln	0		0		3	(0.30%)	0		0	
1	(0.02%)	c.3G>C	p.Met1?*	0		0		0		1	(0.10%)	0	
1	(0.02%)	c.71T>C	p.Leu24Ser	0		0		1	(0.10%)	0		0	
1	(0.02%)	c.131C>T	p.Thr44Met	0		0		0		0		1	(0.10%)
1	(0.02%)	c.275G>A	p.Arg92Gln	0		0		0		0		1	(0.10%)
1	(0.02%)	c.[320C>T; 373A>G]	p.[Thr107Ile; Ile125Val]	1	(0.08%)	0		0		0		0	
1	(0.02%)	c.409T>G	p.Tyr137Asp	1	(0.08%)	0		0		0		0	

Super-populations defined in the 1000 Genomes Project: AFR = African. AMR = Ad Mixed American. EAS = East Asian. EUR = European. SAS = South Asian.

* Start codon lost. Next methionine is found 69bp downstream.

Supplementary Table 8. Relative EMP3 expression compared to CD81 and glycophorin A obtained from proteomics data generated by previous MS analysis of undifferentiated (expanding) BEL-A2 cell line compared with cultured adult erythroid cells at day 8 of culture[6].

Accession	Description	Gene ID	Average Abundances (n=3): cRBC-d8*	Average Abundances (n=3): BELA2	Coverage	# Peptides	# PSMs	# Unique Peptides	# AAs	MW [kDa]	calc. pI	Gene ID
M0R122	Epithelial membrane protein 3 OS=Homo sapiens GN=EMP3 PE=1 SV=1	EMP3	63.7	135.7	59	3	4	3	63	7.1	4.72	
A0A024RCB7	Tetraspanin OS=Homo sapiens GN=CD81 PE=3 SV=1	CD81	82.1	101.8	28	4	6	4	236	25.8	5.29	CD81
A0A0C4DFT7	Glycophorin-A OS=Homo sapiens GN=GYPA PE=1 SV=1	GYPA	102.7	98.6	21	2	5	2	150	16.4	5.27	GYPA
	**Ratio of EMP3/CD81**		**0.78**	**1.33**								
	**Ratio of EMP3/GYPA**		**0.62**	**1.38**								

Supplementary Table 9. Serological testing of human anti-MAM and monoclonal anti-EMP3 with two MAM-negative patients (P8, P9) and two MAM-positive controls (C1, C2).

RBCs	Antibodies							
	Anti-MAM		Anti-EMP3		Anti-Rh		Anti-RhAG	
	Unt	Pap+F	Unt	Pap+F	Unt	Pap+F	Unt	Pap+F
**P8**	0	0	0	0	4+	4+	4+	0
**P9**	0	0	0	0	4+	4+	4+	0
**C1**	4+	4+	0	3+	4+	4+	4+	0
**C2**	4+	4+	0	4+	4+	4+	4+	0

Unt, untreated cells; Pap+F, papain+PNGaseF (2 stage) enzyme-treated cells

Supplementary Table 10. Inhibition of anti-EMP3 binding to MAM, where incubation of MAM-positive papain+PNGaseF treated RBCs with human anti-MAM blocked the binding of murine monoclonal anti-EMP3, whilst no blocking was observed with an unrelated antibody, anti-Jk^b.

	Unsensitised RBCs	Anti-MAM sensitised RBCs	Anti-Jk^b sensitised RBCs
**anti-EMP3**	3+	0	3+
**anti-Rh**	4+	4+	4+
**Isotype control**	0	0	0

Supplementary Table 11. Representative literature assessing EMP3 expression levels in cancers from diverse tissue sources, where *EMP3* has been reported either as an oncogene (upregulated) or as a tumour suppressor (downregulated).

Tissue	EMP3 level	Proliferation	Principal methods	Reference
Uveal melanoma	↑	N/R*	Transcriptomics, gene suppression	Kaochar *et al.*[7]
Glioma	↑	N/R	Microarray, RT-qPCR	Gao *et al.*[8]
Primary breast carcinoma	↑	N/R	miRNA	Hong *et al.*[9]
Hepatocellular carcinoma	↑	+	EMP3 knockdown	Hsieh *et al.*[10]
SKOV3 ovarian cancer cell line	↑	No effect	siRNA	Huang *et al.*[11]
Upper urinary tract urothelial carcinoma	↑	+	siRNA, methylation analysis	Wang *et al.*[12]
Primary breast carcinoma	↑	+	RT-PCR, methylation analysis	Zhou *et al.*[13]
Primary glioblastoma	↑	N/R	Microarray, RT-qPCR	Scrideli *et al.*[14]
Mammary luminal epithelial cells	↑	N/R	Microarray, expression profiling	Mackay *et al.*[15]
Non-small cell lung cancer	↓	N/R	Immunohistochemistry	Xue *et al.*[16]
Oligodendroglioma	↓	N/R	Methylation analysis	Pasini *et al.*[17]
Esophageal squamous cell carcinoma cell line	↓	-	Microarray, methylation analysis	Fumoto *et al.*[18]
Gliomas	↓	N/R	Microarray, methylation analysis	Kunitz *et al.*[19]
Gliomas and neuroblastomas	↓	-	Microarray, methylation analysis	Alaminos *et al.*[20]

* Not reported

Supplementary Table 12. Primers for PCR amplification and Sanger sequencing of *EMP3* and erythroid isoform of *CD44H*.

Amplified Region	Primer*	Primer Sequence (5' to 3')	Primer Position†	PCR Amplification Enzyme & Annealing Temp. (°C)	Product Size (bp)
EMP3 exon 2_3	EMP3 Ex2F<br>EMP3 Ex3R	CGATCTCCTGACCTCAGGTG<br>CCCAATTCTCAGTCCCATGAG	inton 1 (-121 to -102)<br>intron 3 (+78 to +58)	BioTaq, 65	993
EMP3 exon 3	EMP3 Ex3F<br>EMP3 Ex3R	TTACAGGCATGAACCACCAGG<br>As above	intron 2 (-207 to -187)<br>As above	BioTaq, 60	398
EMP3 exon 4	EMP3 Ex4F<br>EMP3 Ex4R	AGGGTCACATGGTGAGCAAG<br>GCAGAGATGTAGCCCACGG	intron 3 (-81 to -62)<br>intron 4 (+162 to +144)	BioTaq, 65	384
EMP3 exon 5	EMP3 Ex5F<br>EMP3 Ex5R	CCATGGGATATTGGGAAATGTAG<br>AGAGGTCAAGCGTGCGCAG	intron 4 (-67 to -45)<br>intron 5 (+62 to +44)	BioTaq, 65	382
EMP3 exon 4 spanning exon 3 to 5	EMP3 Ex3F<br>EMP3 Ex5R	As above<br>As above	As above<br>As above	Expand, 60	3312
EMP3 exon 5 spanning exon 4 to TMEM143	EMP3 Ex4F<br>TMEM143R<br>EMP3 In4F	As above<br>GCCTGATGAACAGAGGAGATAG<br>CCGTGGGCTACATCTCTGC	As above<br>intron 5 (+1524 to +1503)<br>intron 4 (+144 to +162)	Expand, 60<br>Not applicable	2807<br>Sequencing primer
EMP3 complete gene	CCDC114F<br>TMEM143R	CTGTGCTATACTGGTATTCCAG<br>As above	*EMP3* -3949 to *EMP3* -3928<br>As above	BioTaq, 62	9182
CD44 exon 1	CD44_1F<br>CD44_1R	ACTTCCGAGGCAGCCTCATTG<br>ACACAATTCTCCAACGGTTTAGCG	exon 1 (-147 to -127)<br>intron 1 (+156 to +178)	AmpliTaq Gold, 65	392
CD44 exon 2	CD44_2F<br>CD44_2R	TGTTAACCAGGCTGGTCTTGAG<br>AGTTCTAAGCCCAGCTGCCTG	intron 1 (-196 to -175)<br>intron 2 (+48 to +68)	AmpliTaq Gold, 65	430
CD44 exon 3	CD44_3F<br>CD44_3R	TAACTCGGTTGTTGAAACCTCCG<br>AGCTGAGCTCCAAAGACCAGG	intron 2 (-141 to -119)<br>intron 3 (+46 to +66)	AmpliTaq Gold, 60	341
CD44 exon 4	CD44_4F<br>CD44_4R	GCTTCCACAGTGCCTGATATAG<br>TACCACAGAGAACACACCTGAG	intron 3 (-286 to -265)<br>intron 4 (+51 to +72)	AmpliTaq Gold, 60	427
CD44 exon 5	CD44_5F<br>CD44_5R	TCTCCCACCACTGGATAGATAGG<br>GTGCAATGCTCAGGAAGGTCAG	intron 4 (-132 to -110)<br>intron 5 (+55 to +76)	AmpliTaq Gold, 60	439
CD44 exon 15	CD44_15F<br>CD44_15R	GATAGGCTGTATAAGAATGCAAAGG<br>GTGTCAGTATTACCAGGGAACTG	intron 14 (-92 to -68)<br>intron 15 (+223 to +245)	AmpliTaq Gold, 65	400
CD44 exon 16	CD44_16F<br>CD44_16R	GGAGAGCTGCCCTTTATGCAG<br>AGCTAGTTTGCAGAACCCAGG	intron 15 (-86 to -66)<br>intron 16 (+200 to +220)	AmpliTaq Gold, 65	378
CD44 exon 17	CD44_17F<br>CD44_17R	CTGTGGTGCTTGTTTCAACTAGG<br>AGGGACTACGCTCTGAGCAG	intron 16 (-101 to -79)<br>intron 17 (+69 to +88)	AmpliTaq Gold, 60	268

*F and R denote forward (sense) and reverse (antisense) direction, respectively

†Primer positions relative to the respective exon/intron boundary.

Position in *CCDC114* (upstream of *EMP3*) is given relative to the start of *EMP3* coding +1A of ATG codon in exon 2, and in *TMEM143* (downstream) it is relative to the end of *EMP3* exon 5.

Supplementary Table 13. Parameters for homology modelling, molecular dynamics and software packages used.

Method	Model/Parameter	Reference
SWISS-MODEL	Initial automated homology model building	Biasini, M., *et al*.[21]
MODELLER 9.19	Rebuilding of first extracellular loop	Marti-Renom, *et al*.[22]
CHARMM-GUI Membrane Builder	Asymmetric membrane bilayer assembly and homology model insertion, initial thermalisation and equilibration parameters	Wu, E.L., *et al*.[23]
Force field parameters for lipids and protein	CHARMM36<br>Protein<br>Lipids	 Best, R.B., *et al*.[24]<br>Klauda, J.B., *et al*.[25]
Solvation model	TIP3	Jorgensen, W.L., *et al*.[26]
Molecular Dynamics Calculations	NAMD 2.11	Phillips, C.J., *et al*.[27]
VMD (Visual Molecular Dynamics)	MD model assembly and topology file, visualisation. Trajectory analysis	Humphrey, W., *et al*.[28]
MEMBPLUGIN	Membrane analysis, bilayer thickness	Guixà-González, R., *et al*.[29]
PROTTER	Topology representation for homology model	Omasits, U., *et al*.[30]

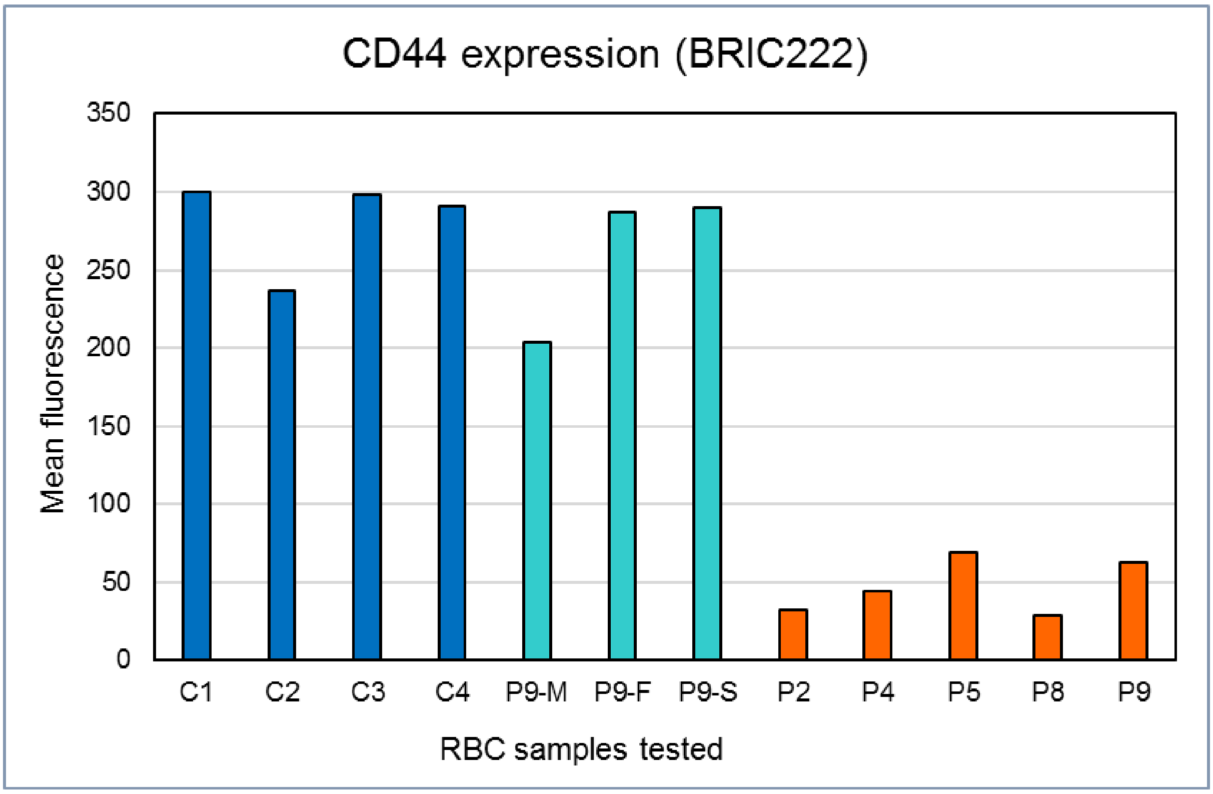

Supplementary Figure 1. Flow cytometry experiments show CD44 to be markedly reduced on RBCs of MAM-negative individuals. Flow cytometric data from RBCs of five MAM-negative individuals [P2, P4, P5, P8, P9], three family members of P9 [mother (P9-M), father (P9-F) and sibling (P9-S)] and four random control samples [C1-C4] tested with anti-CD44 (BRIC222). All MAM-negative samples had a reduction in fluorescent signal compared to the control samples in agreement with serological (Supplementary table 3) and immunoblotting results (shown in Supplementary Figure 7a). The amount of CD44 on MAM-positive RBCs of the parents (F1 and F2) and sibling (F3) of P9, all MAM heterozygotes, had similar expression of CD44 to control samples. Source data are provided as a Source Data file.

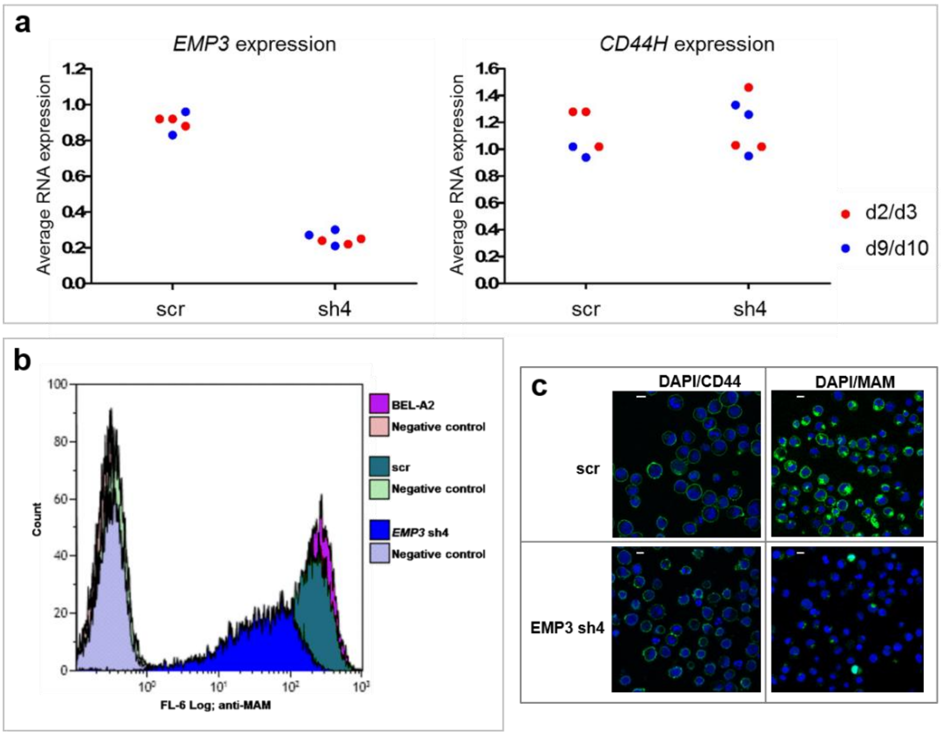

Supplementary Figure 2. Reduction of EMP3 expression examined by cell surface expression analyses and qPCR. Wild-type BEL-A2 cells were maintained in culture for 65 days, with doubling time of 23.9 h. Lentiviral delivery system was used to package and prepare four EMP3 shRNA constructs, of which two worked well (EMP3 sh4 only shown here) and scrambled controls (scr) in HEK293T cell line. BEL-A2 cells were transduced with concentrated virus, and transduced cells were cultured for 28 days following the selection in puromycin. Scrambled control cells retained the same doubling time as native BEL-A2, whilst EMP3 shRNA transduced cells had an improved doubling time of 21.1 h, showing 10-fold higher cumulative increase in cell number on day 28 than the control. **(a)** Reduction in *EMP3* RNA expression (to approximately 25% of scr in EMP3 sh4 cells) was monitored by qPCR on days 2 or 3, and at days 9 or 10 post-transduction. No reduction was observed in *CD44H* RNA expression levels. Source data are provided as a Source Data file. **(b)** Approximately 50% reduction of EMP3 in EMP3 sh4 cells was determined by flow cytometry using a human anti-MAM antibody (P9; prepared by adsorption and elution from antigen-positive cells). **(c)** Confocal microscope immunofluorescence analysis (magnification 40x) showed no difference in CD44 expression between EMP3 sh4 and scr cells when stained with anti-CD44 on day 5 of differentiation. However, when sh4 and scr cells were stained with anti-MAM on day 5 of differentiation, reduction of EMP3 in sh4 cells was observed. Representative images selected from 10 images. The same results were obtained with EMP3 sh2 (not shown). CD44 and MAM stained green; DAPI stained blue. Scale bars are 10 µm.

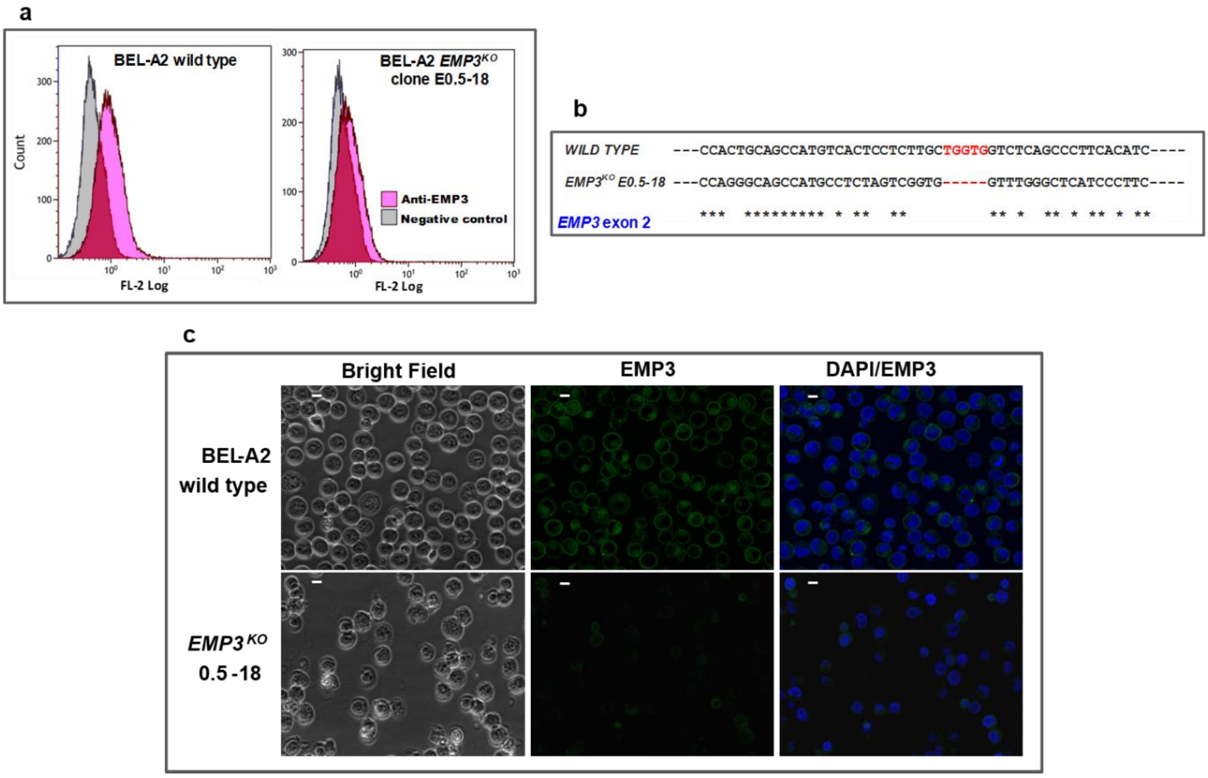

Supplementary Figure 3. Disruption of the *EMP3* gene by CRISPR/Cas9 gene editing in BEL-A2 cell lines; clone E0.5-18 stained with commercially available anti-EMP3. (a) Cells from three of the eight successful transfectant KO clones, as determined by *EMP3* Sanger sequencing and anti-MAM staining, were also tested with anti-EMP3 monoclonal antibody, to assess directly the expression of EMP3 on cell surfaces. Flow cytometry assays displayed reduced expression of EMP3. **(b)** Sanger sequencing of *EMP3* exon 2 confirmed an inactivating deletion in KO clone E0.5-18, NM_001425.2: c.17_21delTGGTG. The deletion, detected close to the location of the guide DNA, would introduce a reading frame shift and premature termination of the protein translation (p.Leu6ArgfsTer49). **(c)** Immunofluorescence assays displayed markedly reduced expression of EMP3. EMP3 stained green; DAPI stained blue. Scale bars are 10 µm. Three KO clones were stained on two different days with anti-EMP3, all giving the same results. In general, commercially available monoclonal anti-EMP3 proved to be poor reagents and a low level of staining of KO cells was observed with both flow cytometry and immunofluorescence methods. We suspect this was due to cross-reactivity with the other members of the EMP family of proteins. A similar level of cross-reactivity was detected by serological tests (data not shown).

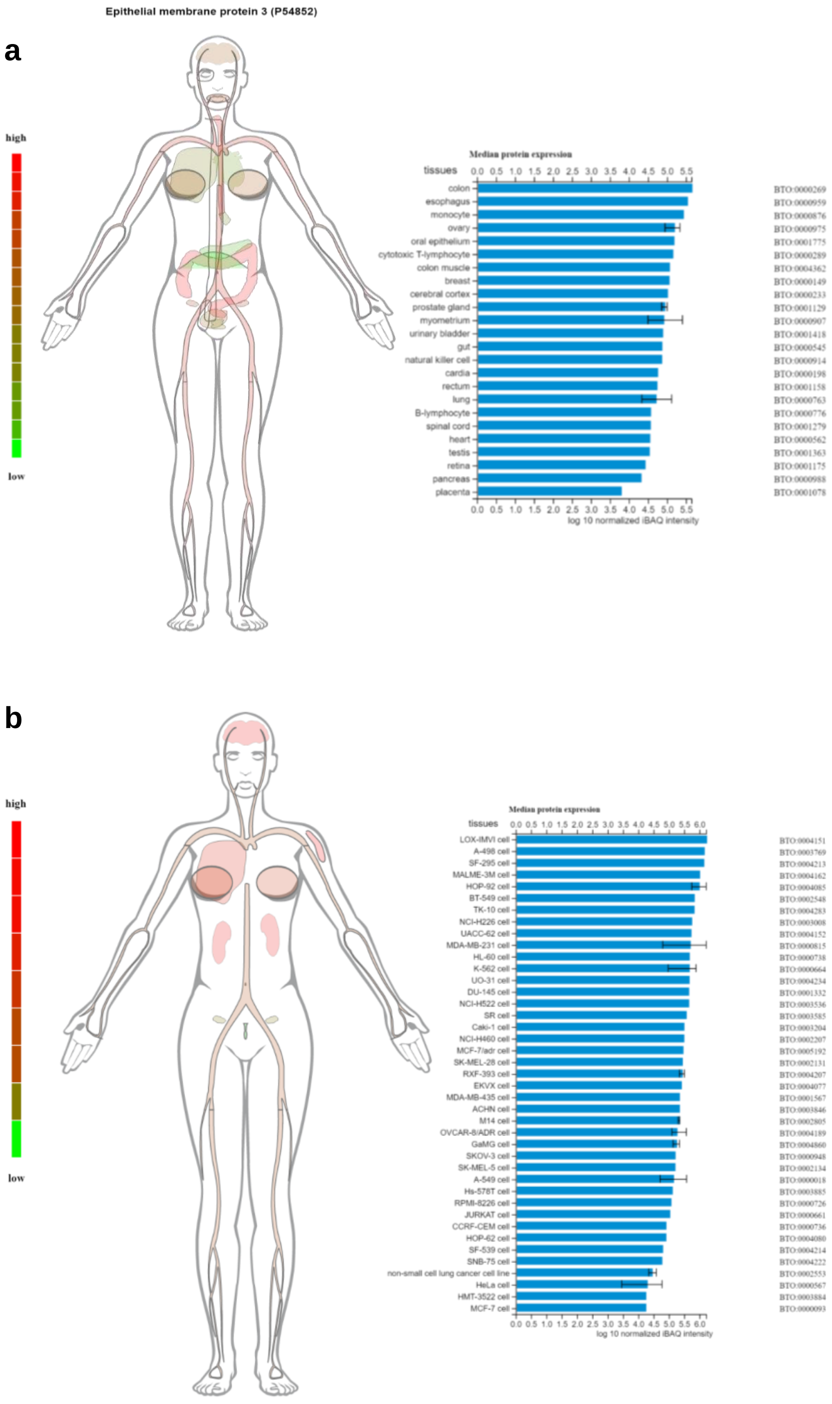

Supplementary Figure 4. EMP3 distribution in human tissues and cell lines. EMP3 expression levels in **a)** human tissues and **b)** a selection of cell lines, shown as median protein expression data from proteomics-based draft of the human proteome, collated by ProteomicsDB[31,32].

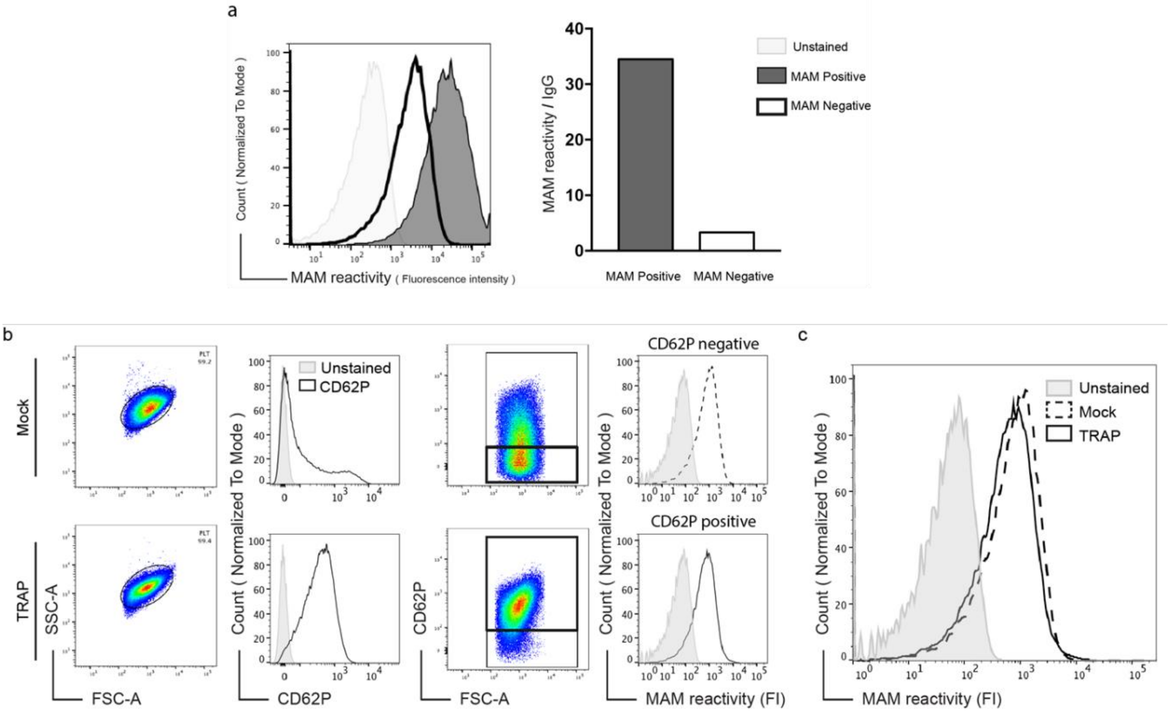

Supplementary Figure 5. MAM antigen is expressed on platelets and is not affected by the platelet activation status. a) The reactivity on platelets of MAM-positive and -negative (P10) samples following incubation with either anti-MAM eluate or isotype control (IgG) is shown as a histogram and a graph of mean fluorescent intensity of MAM reactivity. Source data are provided as a Source Data file. **b)** MAM expression is shown to be independent of the platelet activation status. Representative flow cytometry plots of platelets (collected from leucocyte-depleted apheresis units) and treated with Mock (top panel) and TRAP (bottom panel) were assessed (from left to right) for CD62P expression and further gated on CD62P-negative and -positive to analyse MAM expression. **c)** FACS fluorescence intensity (FI) histogram overlay for MAM expression on non-activated and activated platelets showed no differences in MAM expression.

a

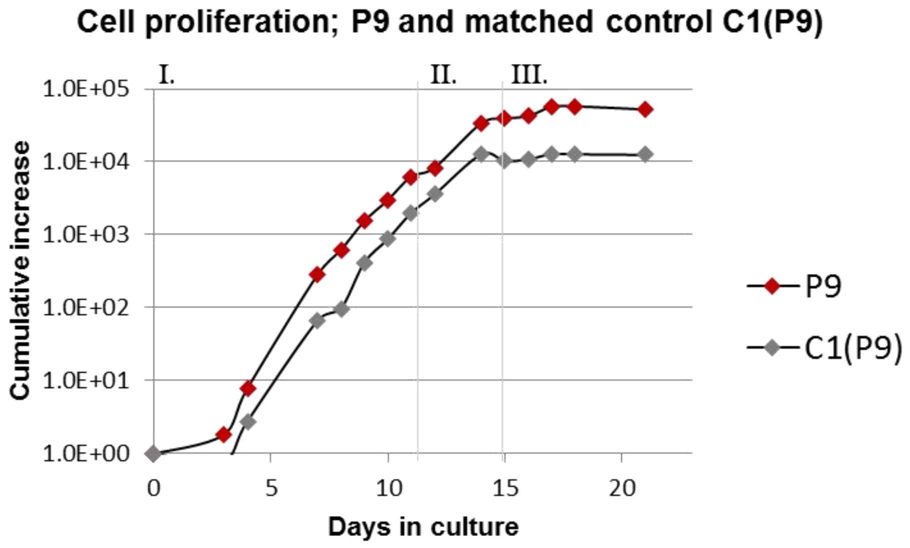

b

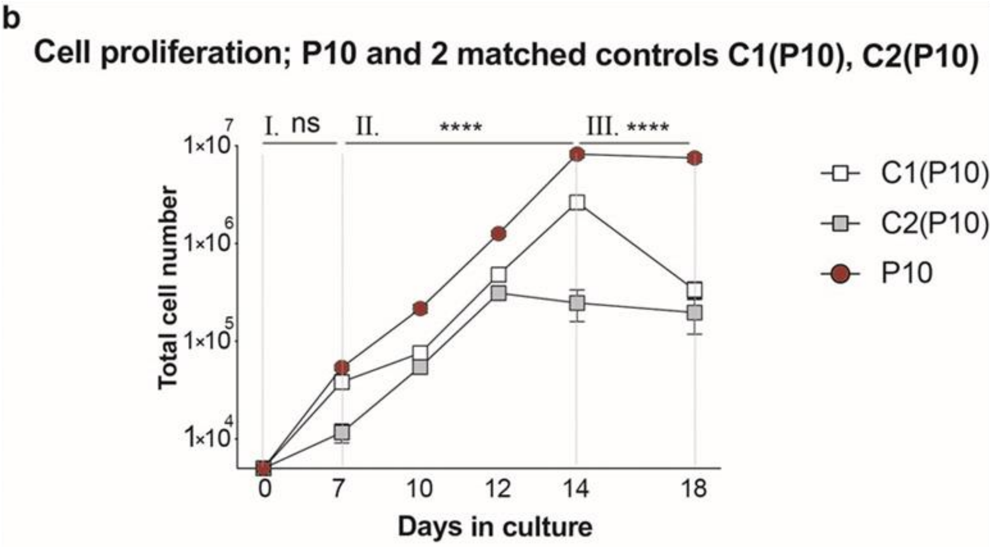

Supplementary Figure 6. Enhanced proliferation of P9 and P10 erythroid cells compared to age- and gender-matched controls, observed in two independent experiments with different culturing protocols, conducted in separate laboratories. CD34+ cells were isolated from buffy coats of P9, P10 and associated controls C1(P9) and C1(P10), C2(P10). **(a)** P9 and C1(P9) cells were cultured at IBGRL (Bristol, UK) for 21 days following the three-stage protocol described in Griffiths *et al*.[33] P9 showed 4.2 times greater cell proliferation on day 21 than C1(P9) control. Source data are provided as a Source Data file. **(b)** P10 and two matched controls C1(P10), C2(P10) were cultured at the Transfusion Medicine laboratory, Lund University (Lund, Sweden) for 18 days, using a different three-stage method based on Giarratana *et al*.[34] and Flygare *et al*.[35] Culture stages labelled I, II, III. The results were analyzed using two-way ANOVA with adjustment for multiple comparisons. Error bars represent SEM of three technical replicates. P10 also had on average four times greater cell proliferation than C1(P10) control. ns, not significant; ****, P<0.0001. Source data are provided as a Source Data file.

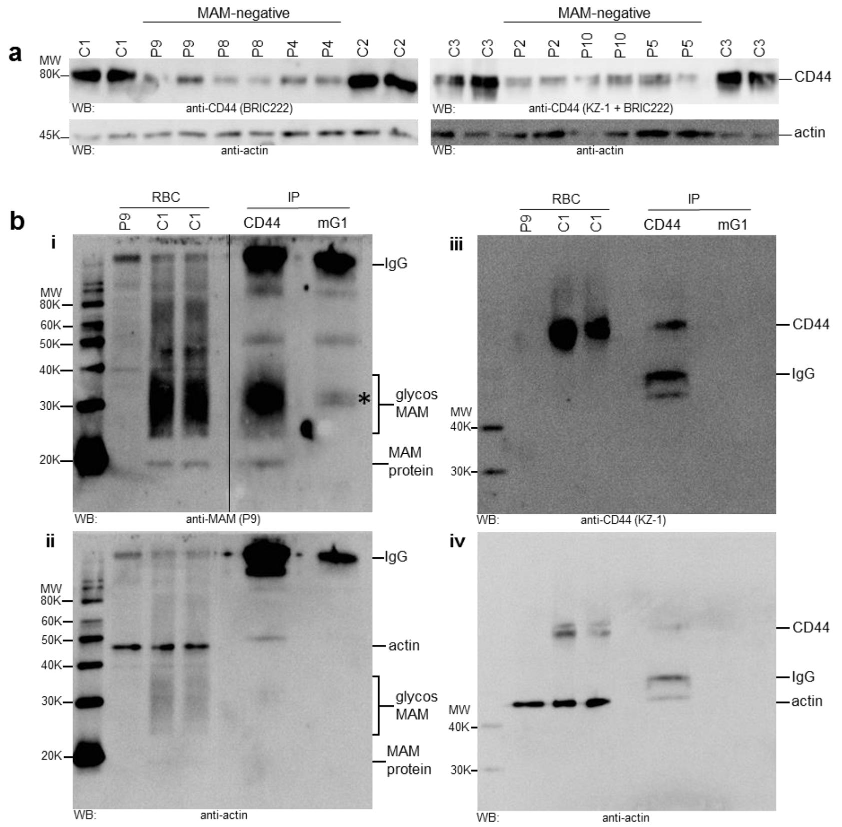

Supplementary Figure 7. Immunoblotting and immunoprecipitation experiments demonstrate the marked reduction of CD44 on RBCs of MAM-negative individuals and the association between erythroid CD44 and the MAM-reactive glycoprotein. a) Immunoblotting of anti-CD44 antibodies to the electrophoretically separated components of RBC membranes from MAM-negative individuals (P2 [n = 3], P4 [n = 2], P5 [n = 3], P8 [n = 3], P9 [n = 4], P10 [n = 3]) and three random controls (C1 [n = 3], C2 [n = 2], C3 [n = 3]). 10% gels, non-reducing conditions, 10 µg protein loaded per lane. MAM-negative RBC membranes have a markedly reduced amount of CD44; the anti-actin control demonstrates consistent protein loading. The numbers quoted are from independent experiments; in 4 out 6 experiments the blots were run in duplicate and gave identical results in both duplicates (P4, n = 3; P2, P5 and P10, n = 6; P8, n = 4; P9 n = 9). Source images are provided in Supplementary Figure 11 and as a Source Data file. **b)** Immunoblotting of human anti-MAM eluate to the electrophoretically separated components of RBC-derived CD44 immune precipitates. **i)** Reactivity of human anti-MAM eluate (P9) with control (C1, the same control as in (a) above) and MAM-negative (P9) RBC membranes and control RBC-derived CD44 (CD44 IP) or isotype control (mG1 IP) immune precipitates electrophoresed on a 12% gel (non-reducing conditions; n = 2). *Reactivity with Protein G that is released in small quantities from the Protein G Sepharose used to isolate the immune complexes. **ii)** The same blot as (i) reprobed with anti-actin to demonstrate equivalent loading of RBC membrane samples (10 µg per lane). **iii)** The positive control gel of RBC-derived CD44 (CD44 IP) or isotype control (mG1 IP) immune precipitates and control and MAM- (C1, P9) RBC membranes immunoblotted with anti-CD44 (10% gel, reducing conditions; n = 2). **iv)** The same blot as (iii) reprobed with anti-actin to demonstrate equivalent loading of RBC membrane samples (10 µg per lane). Source images are provided in Supplementary Figure 11 and as a Source Data file.

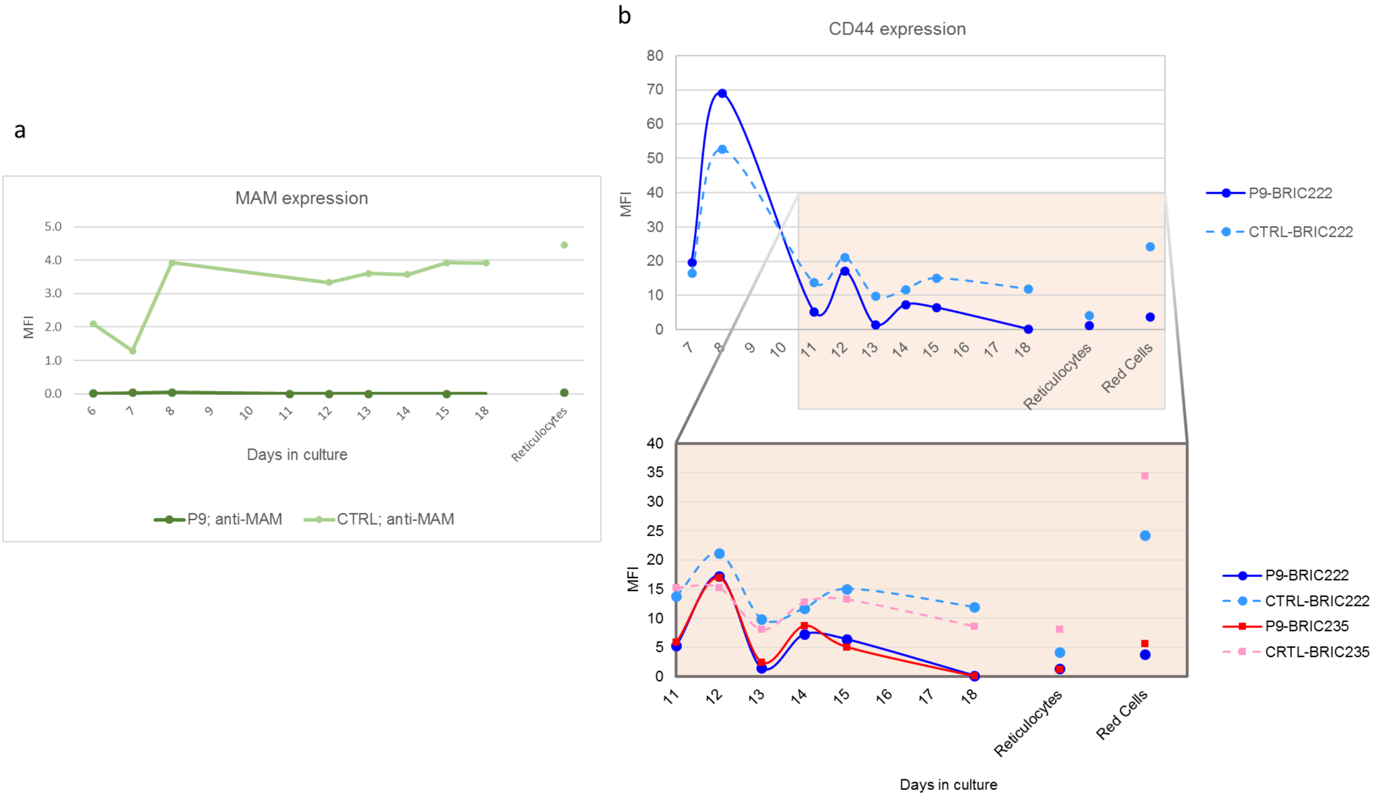

Supplementary Figure 8: MAM (EMP3) and CD44 expression during *ex vivo* cell culture of MAM-negative example (P9) and the matched control (CTRL), as determined by flow cytometry analysis. CD34+ progenitor cells from a buffy coat of P9 and age, gender matched control were cultured for 21 days by three-stage protocol[33]. **a)** Expression levels of cell surface MAM antigen were monitored by immunostaining with anti-MAM eluate from day 6 of the culture. No detectable MAM expression was observed in P9 for the duration of the culture, whilst MAM expression in CTRL increased on day 8 and was subsequently maintained at approximately the same level. Source data are provided as a Source Data file. **b)** Expression levels of cell surface CD44 were followed by immunostaining with monoclonal BRIC222 anti-CD44 from day 7 of the culture. In the later stages of the culture, when cells were more abundant, CD44 expression was examined by an additional monoclonal anti-CD44, BRIC235. CD44 expression was high in the early erythroid cells (day 8 – 10) but it was much reduced in the later stages (day 15 onwards), especially in P9, where no detectable CD44 was seen on cell surfaces on day 18. Filtered reticulocytes and red cells from P9 retained lower levels of CD44, compared to the control cells. Source data are provided as a Source Data file.

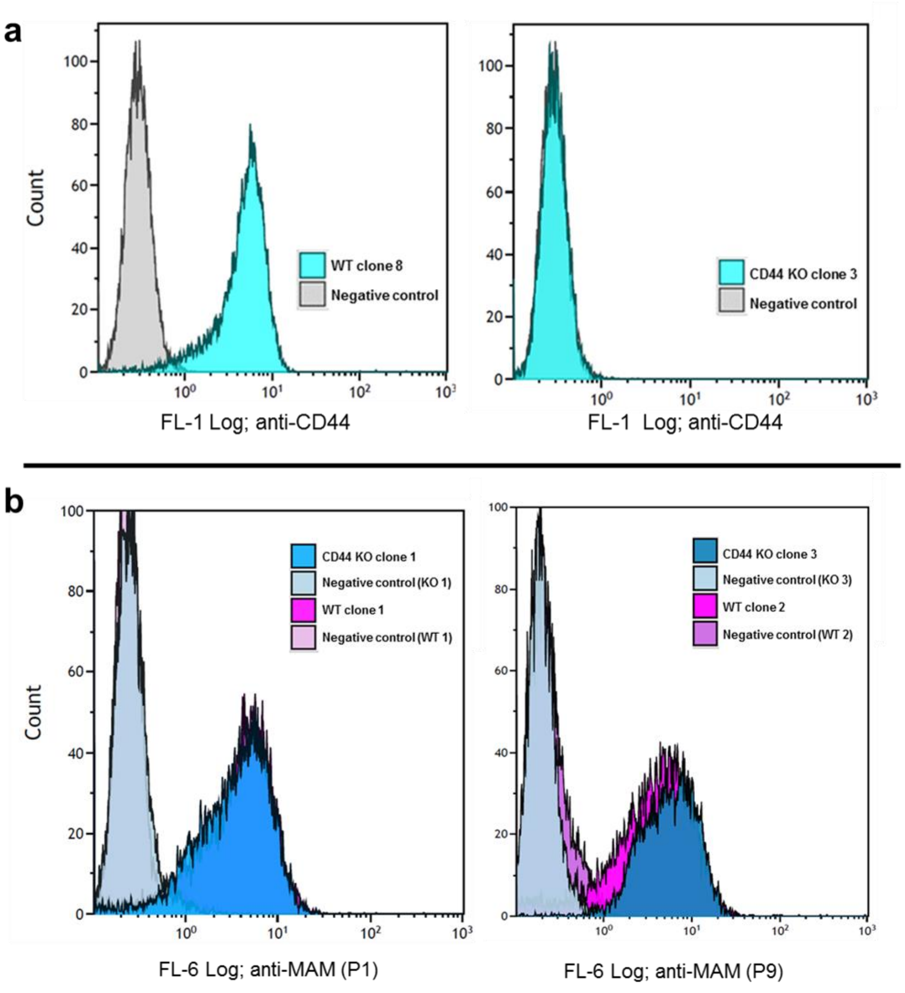

Supplementary Figure 9. The total ablation of CD44 in BEL-A2 cell line does not affect expression of MAM. (a) Flow cytometry analysis of three BEL-A2 wild-type cells treated with an empty vector (WT) and three knock-out (KO) clones with anti-CD44 (BRIC222). The CRISPR/Cas9-mediated gene editing system was used to disrupt CD44 in BEL-A2 erythroid cell line. All three tested transgenic BEL-A2 CD44 KO clones had no detectable CD44 on cell surfaces. The representative histogram shows CD44 reactivity relative to isotype control in CD44 KO clone 3, compared to wild type BEL-A2 cells treated with a mock vector. **(b)** Flow cytometry analysis of BEL-A2 three CD44 KO clones and three wild type clones treated with a mock vector. Representative histograms show anti-MAM reactivity relative to negative control. MAM expression remains the same in CD44 KO clones when tested with two human anti-MAM eluates ($P=0.831$ for P1 and $P=0.719$ for P9 eluate; unpaired two-tailed t-test, $n=6$ for each antibody).

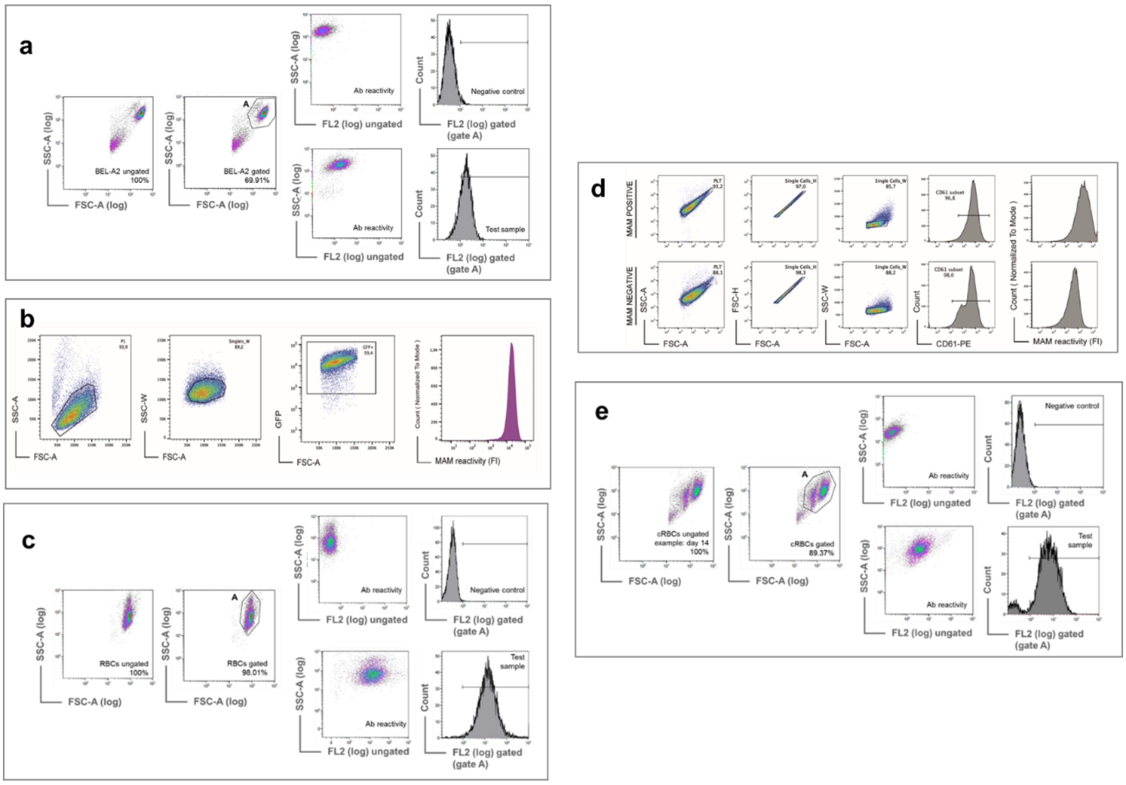

Supplementary Figure 10. The representative gating strategies for flow cytometric evaluation. (a) Gating strategy used in flow cytometry experiments depicted in Figure 2a (analysis of EMP3 KO clone E0.5-6 with anti-MAM), Supplementary Figure 2b (reduction of EMP3 expression in BEL-A2 transduced with EMP3 shRNA), Supplementary Figure 3a (analysis of EMP3 KO clone E0.5-18 with anti-EMP3) Supplementary Figure 9a, b (total ablation of CD44 in BEL-A2 cell line does not affect expression of MAM); these experiments utilised BEL-A2 cells, all analysed by the same gating strategy. The gate on the FSC vs SSC dot plot defines the cellular population; the isotype negative control was used to set the FL-2 or FL-6 fluorescence voltages such that the gated cells fell within the first decade. **(b)** Figure 3 (*EMP3* transfection causes MAM expression in Daudi cells); live Daudi cells were identified and gated; after doublet exclusion the GFP-positive cells were identified and MAM reactivity was measured on this third gated cell population. **(c)** Supplementary Figure 1 (CD44 is markedly reduced on RBCs of MAM-negative individuals); the gate on the FSC vs SSC dot plot defines the red cell population; the isotype negative control was used to set the FL-2 fluorescence voltages such that the gated cells fell within the first decade. All samples were analysed by the same gating protocol. **(d)** Supplementary Figure 5 (MAM antigen is expressed on platelets and is not affected by the platelet activation status); platelets isolated from leucocyte-depleted apheresis units were gated on CD61-positivity after doublet exclusion, then MAM reactivity checked within the CD61-positive cells. **(e)** Supplementary Figure 8a, b (MAM and CD44 expression during *ex vivo* cell culture of MAM-negative (P9) and control samples); cultured red cells (cRBCs) were analysed on days 6 to 18 of culture. The gate on the FSC vs SSC dot plot defines the cellular population; the isotype negative control was used to set the FL-1, FL-2 or FL-6 fluorescence voltages such that the gated cells fell within the first decade. Anti-MAM and anti-CD44 expression were measured on the gated population of cells compared to their respective negative controls.

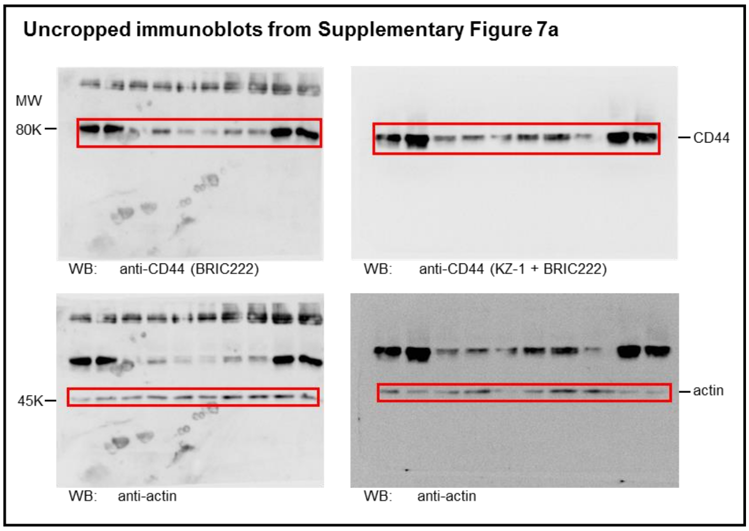

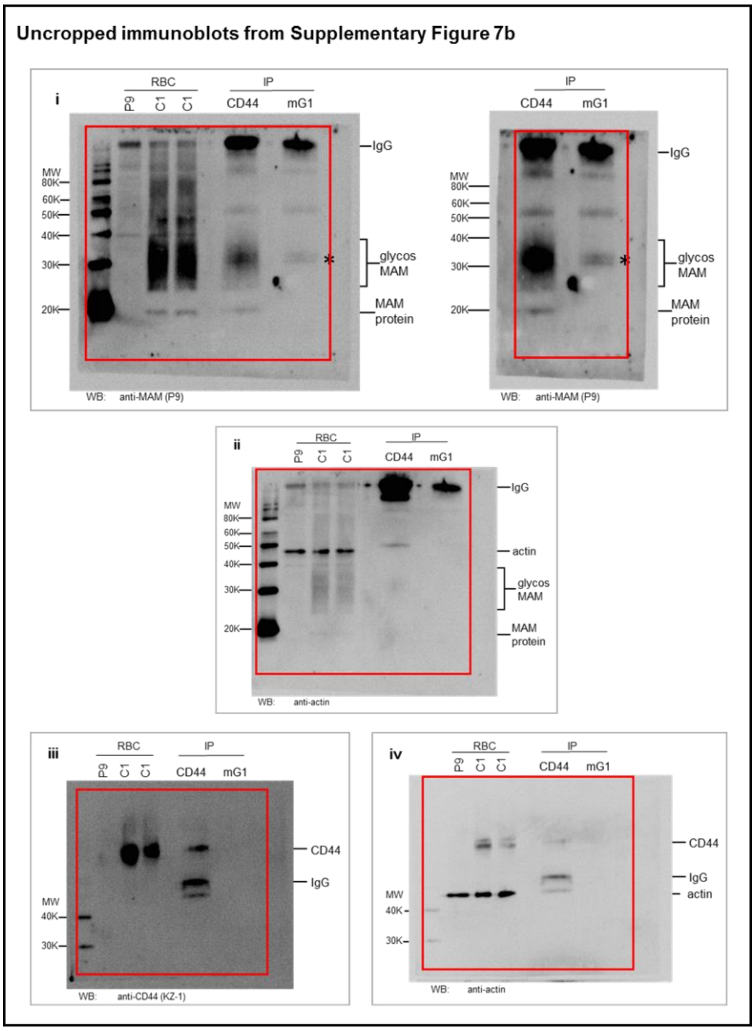

Supplementary Figure 11. Uncropped original immunoblotting scans from Supplementary Figure 7. Immunoblots presented in Supplementary Figure 7a, b are marked here by the red outline.

Supplementary References

1. Anderson, G., Donnelly, S., Brady, T., Mintz, P. D., Sinor, L. and Daniels, G.L. An antibody to a high frequency antigen found on red cells, platelets, lymphocytes, and monocytes. *Transfusion* **33**(**9**): 23S (1993).
2. Montgomery, W., Nance, S. J., Donnelly, S. F., Brady, T. W., Anderson, G., Mintz, P. D., Moulds, M. K., Daniels, G. L., Spring, F. A., Molina, N., de Asis, E. A., Olivares, E. MAM: a “new” high-incidence antigen found on multiple cell lines. *Transfusion* 40: 1132-1139 (2000).
3. Burgos, A., Martinez, V., Velliquette, R. W., Ariza, E., Sylvestre, G., Lomas-Francis, C., Westoff, C. M. First report of anti-MAM in pregnancy without evidence of clinical HDFN of thrombocytopenia. *Transfusion* 52 (suppl): 26A (2012).
4. Lek, M., Karczewski, K.J., Minikel, E.V., Samocha, K.E., Banks, E., *et al*. Analysis of protein-coding genetic variation in 60,706 humans. *Nature* **536**: 285–291 (2016).
5. The 1000 Genomes Project Consortium. A global reference for human genetic variation. *Nature* **526**: 68–74 (2015).
6. Trakarnsanga, K., Griffiths, R.E., Wilson, M.C., Blair, A., Satchwell, T.J., Meinders, M., Cogan, N., Kupzig, S., Kurita, R., Nakamura, Y., Toye, A.M., Anstee, D.J. and Frayne, J. An immortalized adult human erythroid line facilitates sustainable and scalable generation of functional red cells. Nat. Commun. 8: 14750 (2017).
7. Kaochar, S., Dong, J., Torres, M., Rajapakshe, K., Nikolos, F., Davis, C.M., Ehli, E.A., Coarfa, C., Mitsiades, N. and Poulaki, V. ICG-001 Exerts Potent Anticancer Activity Against Uveal Melanoma Cells. *Invest Ophthalmol Vis Sci.* **59**(**1**): 132-143 (2018).
8. Gao, Y.F., Zhu, T., Mao, C.X., Liu, Z.X., Wang, Z.B., Mao, X.Y., Li, L., Yin, J.Y., Zhou, H.H. and Liu, Z.Q. PPIC, EMP3 and CHI3L1 Are Novel Prognostic Markers for High Grade Glioma. *Int J Mol Sci.* **17**(**11**), (2016).
9. Hong, X.C., Fen, Y.J., Yan, G.C., Hong, H., Yan, C.H., Bing, L.W. and Zhong, Y.H. Epithelial membrane protein 3 functions as an oncogene and is regulated by microRNA-765 in primary breast carcinoma. *Mol Med Rep.* **12**(**5**): 6445-6450 (2015).
10. Hsieh, Y.H., Hsieh, S.C., Lee, C.H., Yang, S.F., Cheng, C.W., Tang, M.J., Lin, C.L., Lin, C.L. and Chou, R.H. Targeting EMP3 suppresses proliferation and invasion of hepatocellular carcinoma cells through inactivation of PI3K/Akt pathway. *Oncotarget.* **6**(**33**):34859-34874 (2015).
11. Huang, R.Y., Kuay, K.T., Tan, T.Z., Asad, M., Tang, H.M., Ng, A.H., Ye, J., Chung, V.Y. and Thiery J.P. Functional relevance of a six mesenchymal gene signature in epithelial-mesenchymal transition (EMT) reversal by the triple angiokinase inhibitor, nintedanib (BIBF1120). *Oncotarget* **6**(**26**): 22098-22113 (2015).
12. Wang, Y.W., Li, W.M., Wu, W.J., Chai, C.Y., Liu, H.S., Lai, M.D. and Chow, N.H. Potential significance of EMP3 in patients with upper urinary tract urothelial carcinoma: crosstalk with ErbB2-PI3K-Akt pathway. *J Urol.* **192**(**1**): 242-251 (2014).
13. Zhou, W., Jiang, Z., Li, X., Xu, F., Liu, Y., Wen, P., Kong, L., Hou, M. and Yu, J. EMP3 overexpression in primary breast carcinomas is not associated with epigenetic aberrations. *J Korean Med Sci* **24**(**1**): 97-103 (2009).
14. Scrideli, C.A., Carlotti, C.G. Jr., Okamoto, O.K., Andrade, V.S., Cortez, M.A., Motta, F.J., Lucio-Eterovic, A.K., Neder, L., Rosemberg, S., Oba-Shinjo, S.M., Marie, S.K. and Tone, L.G. Gene expression profile analysis of primary glioblastomas and non-

neoplastic brain tissue: identification of potential target genes by oligonucleotide microarray and real-time quantitative PCR. *J Neurooncol.* **88**(**3**): 281-291 (2008).

15. Mackay, A., Jones, C., Dexter, T., Silva, R.L., Bulmer, K., Jones, A., Simpson, P., Harris, R.A., Jat, P.S., Neville, A.M., Reis, L.F., Lakhani, S.R., O'Hare, M.J. cDNA microarray analysis of genes associated with ERBB2 (HER2/neu) overexpression in human mammary luminal epithelial cells. *Oncogene* **22**(**17**): 2680-2688 (2003).
16. Xue, Q., Zhou, Y., Wan, C., Lv, L., Chen, B., Cao, X., Ju, G., Huang, Y., Ni, R. and Mao, G. Epithelial membrane protein 3 is frequently shown as promoter methylation and functions as a tumor suppressor gene in non-small cell lung cancer. *Exp Mol Pathol.* **95**(**3**): 313-318 (2013).
17. Pasini, A., Iorio, P., Bianchi, E., Cerasoli, S., Cremonini, A.M., Faedi, M., Guarnieri, C., Guiducci, G., Riccioni, L., Molinari, C., Rengucci, C., Calistr, D. and Giordano, E. LOH 19q indicates shorter disease progression-free interval in low-grade oligodendrogliomas with EMP3 methylation. *Oncol Rep* **28**(**6**): 2271-2277 (2012).
18. Fumoto, S., Hiyama, K., Tanimoto, K., Noguchi, T., Hihara, J., Hiyama, E., Noguchi, T. and Nishiyama, M. EMP3 as a tumor suppressor gene for esophageal squamous cell carcinoma. *Cancer Lett.* **274**(**1**): 25-32 (2009).
19. Kunitz, A., Wolter, M., van den Boom, J., Felsberg, J., Tews, B., Hahn, M., Benner, A., Sabel, M., Lichter, P., Reifenberger, G., von Deimling, A. and Hartmann, C. DNA hypermethylation and aberrant expression of the EMP3 gene at 19q13.3 in Human Gliomas. *Brain Pathol.* **17**(**4**):363-370 (2007).
20. Alaminos, M., Dávalos, V., Ropero, S., Setién, F., Paz, M.F., Herranz, M., Fraga, M.F., Mora, J., Cheung, N-K. V., Gerald, W.L. and Esteller, M. EMP3, a myelin-related gene located in the critical 19q13.3 region, is epigenetically silenced and exhibits features of a candidate tumor suppressor in glioma and neuroblastoma. *Cancer Res.* **65**: 2565-2571 (2005).
21. Biasini, M., Bienert, S., Waterhouse, A., Arnold, K., Studer, G., Schmidt, T., Kiefer, F., Cassarino, T.G., Bertoni, M., Bordoli, L., Schwede, T. SWISS-MODEL: modelling protein tertiary and quaternary structure using evolutionary information. *Nucleic Acids Res.* **42**: W252-W258 (2014).
22. Marti-Renom, M.A., Stuart, A., Fiser, A., Sánchez, R., Melo, F. and Sali, A. Comparative protein structure modeling of genes and genomes. *Annu Rev Biophys Biomo. Structure* **29**: 291-325 (2000).
23. Wu, E.L., Cheng, X., Jo, S., Rui, H., Song, K.C., Dávila-Contreras, E.M., Qi, Y., Lee, J., Monje-Galvan, V., Venable, R.M., Klauda, J.B. and Im, W. CHARMM-GUI Membrane Builder Toward Realistic Biological Membrane Simulations. *J Comput Chem.* **35**: 1997-2004 (2014).
24. Best, R.B., Zhu, X., Shim, J., Lopes, P.E., Mittal, J., Feig, M. and Mackerell, A.D. Jr. Optimization of the additive CHARMM all-atom protein force field targeting improved sampling of the backbone φ, ψ and side-chain χ(1) and χ(2) dihedral angles. *J Chem Theory Comput.* **8**: 3257-3273 (2012).
25. Klauda, J.B., Venable, R.M., Freites, J.A., O'Connor, J.W., Tobias, D.J., Mondragon-Ramirez, C., Vorobyov, I., MacKerell, A.D. Jr. and Pastor, R.W. Update of the CHARMM all-atom additive force field for lipids: validation on six lipid types. *J Phys Chem B.* **114**: 7830-43 (2010).
26. Jorgensen, W.L., Chandrasekhar, J., Madura, J.D., Impey, R.W. and Klein, M.L. Comparison of simple potential functions for simulating liquid water. *J Chem Phys.* **79**: 926–935 (1983).

27. Phillips, C.J., Braun, R., Wang, W., Gumbart, J., Tajkhorshid, E., Villa, E., Chipot, C., Skeel, R.D., Kale, L. and Schulten, K. Scalable molecular dynamics with NAMD. *J Comp Chem.* **26**: 1781-1802 (2005).
28. Humphrey, W., Dalke, W. and Schulten, K. VMD: visual molecular dynamics. *J Mol Graph.* **14**: 33–38 (1996).
29. Guixà-González, R., Rodriguez-Espigares, I., Ramírez-Anguita, J.M., Carrió-Gaspar, P., Martinez-Seara, H., Giorgino, T. and Selent, J. MEMBPLUGIN: studying membrane complexity in VMD. *Bioinformatics* **30**:1478-80 (2014).
30. Omasits, U., Ahrens, C.H., Müller, S. and Wollscheid, B. Protter: interactive protein feature visualization and integration with experimental proteomic data. *Bioinformatics* **30**: 884-886 (2014).
31. Schmidt, T., Samaras, P., Frejno, M., Gessulat, S., Barnert, M., Kienegger, H., Krcmar, H., Schlegl, J., Ehrlich, H.C., Aiche, S., Kuster, B. and Wilhelm, M. ProteomicsDB. *Nucleic Acids Res*. **46**(**D1**): D1271-D1281 (2018).
32. Wilhelm, M., Schlegl, J., Hahne, H., Gholami, A.M., Lieberenz, M., Savitski, M.M., Ziegler, E., Butzmann, L., Gessulat, S., Marx, H., Mathieson, T., Lemeer, S., Schnatbaum, K., Reimer, U., Wenschuh, H., Mollenhauer, M., Slotta-Huspenina, J., Boese, J.H., Bantscheff, M., Gerstmair, A., Faerber, F. and Kuster, B. Mass-spectrometry-based draft of the human proteome. *Nature* **509**(**7502**):582-587 (2014).
33. Griffiths, R.E., Kupzig, S., Cogan, N., Mankelow, T.J., Betin, V.M., Trakarnsanga, K., Massey, E.J., Lane, J.D., Parsons, S.F. and Anstee, D.J. Maturing reticulocytes internalize plasma membrane in glycophorin A-containing vesicles that fuse with autophagosomes before exocytosis. *Blood* **119**(**26**): 6296-6306 (2012).
34. Giarratana, M.C., Kobari, L., Lapillonne, H., Chalmers, D., Kiger, L., Cynober, T., Marden, M.C., Wajcman, H. and Douay, L. Ex vivo generation of fully mature human red blood cells from hematopoietic stem cells. Nat. Biotechnol. 23(1): 69-74 (2005).
35. Flygare, J., Rayon Estrada, V., Shin, C., Gupta, S. and Lodish, H.F. HIF1alpha synergizes with glucocorticoids to promote BFU-E progenitor self-renewal. *Blood* **117**(**12**): 3435-44 (2011).

End of document
