# Supplementary material for: Disruption of the tumour-associated EMP3 enhances erythroid proliferation and causes the MAM-negative phenotype
Source: Nat Commun. 2020 Jul 16;11:3569. doi: 10.1038/s41467-020-17060-4 (PMC7366909; doi:10.1038/s41467-020-17060-4)
Supplement: Supplementary file 2 — Description of Additional Supplementary Files [file 41467_2020_17060_MOESM2_ESM.pdf]

Title: Supplementary Data File 1

Description: The proteomic analysis of immunoprecipitated erythrocyte-derived CD44 demonstrating the association between CD44 and EMP3.
