# Supplementary material for: Disruption of the tumour-associated EMP3 enhances erythroid proliferation and causes the MAM-negative phenotype
Source: Nat Commun. 2020 Jul 16;11:3569. doi: 10.1038/s41467-020-17060-4 (PMC7366909; doi:10.1038/s41467-020-17060-4)
Supplement: Supplementary file 5 — Reporting Summary [file 41467_2020_17060_MOESM5_ESM.pdf]

nature research

Corresponding author(s): Nicole Thornton

Last updated by author(s): Apr 15, 2020

Reporting Summary

Nature Research wishes to improve the reproducibility of the work that we publish. This form provides structure for consistency and transparency in reporting. For further information on Nature Research policies, see Authors & Referees and the Editorial Policy Checklist .

Statistics

For all statistical analyses, confirm that the following items are present in the figure legend, table legend, main text, or Methods section.

n/a	Confirmed	
☐	☒	The exact sample size (*n*) for each experimental group/condition, given as a discrete number and unit of measurement
☐	☒	A statement on whether measurements were taken from distinct samples or whether the same sample was measured repeatedly
☐	☒	The statistical test(s) used AND whether they are one- or two-sided<br>*Only common tests should be described solely by name; describe more complex techniques in the Methods section.*
☒	☐	A description of all covariates tested
☒	☐	A description of any assumptions or corrections, such as tests of normality and adjustment for multiple comparisons
☐	☒	A full description of the statistical parameters including central tendency (e.g. means) or other basic estimates (e.g. regression coefficient) AND variation (e.g. standard deviation) or associated estimates of uncertainty (e.g. confidence intervals)
☐	☒	For null hypothesis testing, the test statistic (e.g. *F*, *t*, *r*) with confidence intervals, effect sizes, degrees of freedom and *P* value noted<br>*Give P values as exact values whenever suitable.*
☒	☐	For Bayesian analysis, information on the choice of priors and Markov chain Monte Carlo settings
☒	☐	For hierarchical and complex designs, identification of the appropriate level for tests and full reporting of outcomes
☒	☐	Estimates of effect sizes (e.g. Cohen's *d*, Pearson's *r*), indicating how they were calculated

Our web collection on statistics for biologists contains articles on many of the points above.

Software and code

Policy information about availability of computer code

Data collection	Whole exome sequence data: MiSeq Reporter v.2.5.1 (Illumina)<br>Flow cytometry acquisition: Navios System Software (Beckman Coulter), BD FACSDiva (Becton Dickinson)
Data analysis	Whole exome sequence analysis: MiSeq Reporter v2.5.1 (Illumina), Variant Studio v2.2 (Illumina), Integrative Genomics Viewer v2.3 (Broad Institute); Sanger sequence analysis: SeqScape Software v.3 (Applied Biosystems); flow cytometry analysis: Kaluza v.1.2 and v.2.1 (Beckman Coultard); BD FACSDiva (Becton Dickinson), FlowJo v.10.4.2; immunoblotting: Kodak 4000R imaging software (Kodak); confocal microscopy: Leica LAS AF software (Leica), Adobe Photoshop CC (Adobe), Volocity v.6.3 (Perkin Elmer); protein modelling: SWISS-MODEL (University of Basel), MODELLER v.9.19 (University of California San Francisco), CHARMM Membrane Builder (CHARMM-GUI), NAMD 2.11 (University of Illinois), VMD (University of Illinois), PROTTER (ETH Zurich), MEMBPLUGIN (SourceForge)

For manuscripts utilizing custom algorithms or software that are central to the research but not yet described in published literature, software must be made available to editors/reviewers. We strongly encourage code deposition in a community repository (e.g. GitHub). See the Nature Research guidelines for submitting code & software for further information.

Data

Policy information about availability of data

All manuscripts must include a data availability statement. This statement should provide the following information, where applicable:

- Accession codes, unique identifiers, or web links for publicly available datasets
- A list of figures that have associated raw data
- A description of any restrictions on data availability

Nucleotide sequence data are available in the DDBJ/EMBL/GenBank databases under the accession numbers MN175570, MN175569, MN164487, MN164486 and MN121937. The source data underlying Figs 3b, 5a, 5c and 6b and Supplementary Figs 1, 2a, 5a, 6a-b, 7a-b and 8a-b are provided as a Source Data file. All other data supporting the findings of this study are available from the corresponding author upon reasonable request.

Field-specific reporting

Please select the one below that is the best fit for your research. If you are not sure, read the appropriate sections before making your selection.

- [x] Life sciences
- [ ] Behavioural & social sciences
- [ ] Ecological, evolutionary & environmental sciences

For a reference copy of the document with all sections, see nature.com/documents/nr-reporting-summary-flat.pdf

Life sciences study design

All studies must disclose on these points even when the disclosure is negative.

Sample size

Number of samples was simply as many MAM-negative samples as we could include. In the end, we have included all MAM-negative samples known through collaboration with different laboratories in the world. Even if other MAM-negative samples no doubt exist, e.g. based on the frequency numbers from the gnomAD database presented in the paper, they are currently not identified as MAM-negative, and therefore we believe all known examples are included in our study. MAM-positive controls were included as appropriate with respect to the experiments undertaken (serological and genetic analysis, cell culture, gene editing, etc.). Wherever possible, for example, for cell culture, we have used age and sex-matched samples collected at the same time and transported under the same conditions. In other tests, such as serological tests, single examples of MAM-positive RBCs were selected as a control, since the purpose was to confirm that the reagent antibody was reactive under the test conditions. For genetic analysis, data obtained from whole exome sequencing and Sanger sequencing was compared to reference sequences and public databases which represent the common MAM-positive phenotype, i.e. an intact EMP3 gene.

Data exclusions

No data were excluded.

Replication

All data were replicated at least twice , e.g. re-sequencing gave the same results, erythroid culture was performed in two laboratories (Bristol and Lund), flow cytometric analyses were repeated as indicated etc. All replications gave the same results.

Randomization

No randomization involved in this study design. This study was initially a discovery project that sought to understand the genetic basis of a well-defined erythrocyte phenotype. Thus randomisation was not appropriate. Covariates in cell culture experiments were controlled by selecting age and sex matched MAM-positive controls. In all experiments MAM-positive and MAM-negative samples were treated identically to eliminate confounding experimental factors.

Blinding

Although blinding was not appropriate or attempted for most experiments in this study, initial cell cultures including MAM-negative and matched control samples were blinded. Where experiments were not blinded, all outcome data was analysed objectively or by software.

Reporting for specific materials, systems and methods

We require information from authors about some types of materials, experimental systems and methods used in many studies. Here, indicate whether each material, system or method listed is relevant to your study. If you are not sure if a list item applies to your research, read the appropriate section before selecting a response.

Materials & experimental systems

n/a	Involved in the study	
[ ]	[x]	Antibodies
[ ]	[x]	Eukaryotic cell lines
[x]	[ ]	Palaeontology
[x]	[ ]	Animals and other organisms
[ ]	[x]	Human research participants
[x]	[ ]	Clinical data

Methods

n/a	Involved in the study	
[x]	[ ]	ChIP-seq
[ ]	[x]	Flow cytometry
[x]	[ ]	MRI-based neuroimaging

Antibodies

Antibodies used

Primary antibodies used in serology, flow cytometry and confocal microscopy were anti-EMP3 (clone 439949, R&D Systems, catalog no. MAB4505); anti-Rh (IBGRL Research Products, product code 9423), anti-RhAG (in-house IBGRL research reagent), anti-CD44 (clones BRIC222, KZ1 and BRIC235, IBGRL Research Products, product codes 9406, 9430 and 9407), anti-Jkb (Lorne Laboratories UK, code 776002); human derived anti-MAM in plasma and eluate form (in-house IBGRL reference collection); mouse anti-human CD62P (P-Selectin, clone AK4; PE conjugated, BioLegend, catalogue no. 304905, diluted 1:100). Secondary antibodies for serology were BIOSCOT® anti-IgG/C3d, polyspecific anti-human globulin blood grouping reagent (Millipore, catalogue no. TS-10X10ML-U) and polyclonal rabbit anti-mouse immunoglobulin G (Dako, catalogue no. Z025902-2).

For flow cytometry, the following secondary antibody conjugates were used: Alexa Fluor® 647-AffiniPure F(ab')2 fragment goat anti-human IgG (Jackson ImmunoResearch Europe, catalogue no. 109-606-006-JIR, diluted 1:100), R-phycoerythrin conjugated polyclonal rabbit anti-mouse IgG,/rabbit F(ab')2, RPE (Dako, catalogue no. R043901-2, diluted 1:20), fluorescein isothiocyanate conjugated polyclonal rabbit anti-mouse IgG/rabbit F(ab')2 FITC (Dako, catalogue no. F031302-2, diluted 1:20) and allophycocyanin (APC)-conjugated AffiniPure F(ab')2 fragment goat-anti-Human IgG (Jackson ImmunoResearch Europe,

catalogue no. 109-136-170, diluted 1:200).

For MAIEA, mouse monoclonal IgG antibodies were used: BRIC222 (product code 9406), BRIC241, BRIC205, BRIC214, BRIC219, BRIC223, BRIC225, BRIC235 (product code 9407), KZ1 (product code 9430), BRIC224 (product code 9427), BRIC221 (product code 9438), BRIC18 (product code 9440), BRIC203 (product code 9425), LA1818, AE-1, BRIC230 (product code 9428), BRIC110 (product code 9402), BRIC5 (product code 9405), BRIC229 (product code 9409), BRIC6 (product code 9439), BIRMA84b (product code 9455), BRIC125 (product code 9454); all IBGRL Research Products or in-house research reagents; HIM6 (BioLegend, catalogue no. 306202), UM-8D6 (Abcam, catalogue no. ab194401), MEM-M6/6 (Abcam, catalogue no. ab119114) and MIMA123 (gift from NYBC)]. Plates were coated with AffiniPure Goat Anti-Mouse IgG, Fcγ fragment specific (Jackson ImmunoResearch Europe, catalogue no. 115-005-071, diluted 1:1000) and complexes were detected with conjugated polyclonal Fcγ fragment-specific goat anti-human peroxidase conjugated IgG (Stratech Scientific Ltd., catalogue no. E-AB-1040-ELA).

For immunoblotting, anti-beta actin antibody (Abcam, clone AC-15, catalogue no. ab6276) was used. Secondary antibodies were alkaline phosphatase-conjugated AffiniPure F(ab')2 fragments of goat anti-mouse IgG1, Fcγ subclass 1 specific and goat anti-human IgG (Jackson ImmunoResearch Europe, catalogue no. 115-055-205 and 109-056-006).

Secondary antibodies for confocal microscopy were goat anti-mouse IgG (H+L) cross-adsorbed secondary antibody, Alexa Fluor 488 and goat anti-rabbit IgG (H+L) cross-adsorbed secondary antibody, Alexa Fluor 546 (Invitrogen, catalogue no. 488A-11001 and A-11010).

Validation

The validation, relevant citations and antibody profiles for the IBGRL Research Products antibodies can be found via their product sheets at https://ibgrl.blood.co.uk/ibgrl-research-products/by-product-code/. For example, KZ-1, BRIC222 and BRIC235 were validated at the IVth Leucocyte Typing Workshop (KZ-1 and B235) and by epitope mapping with known workshop Mabs and published in Anstee et al 1991 (Immunology). BRIC241, BRIC205, BRIC214, BRIC219, BRIC223, BRIC225, LA1818, AE-1, MIMA123 are not commercially available, but they have been validated by standard serological tests at IBGRL, Bristol and NYBC, New York. Human anti-MAM eluate was prepared by adsorption and acid elution method from MAM-positive cells and validated by standard serological tests at IBGRL, Bristol.

Anti-Jkb (https://www.lornelabs.com/products/blood-transfusion/blood-grouping-reagents/anti-jkb-polyclonal), HIM6 (https://www.biolegend.com/en-us/products/purified-anti-human-cd147-antibody-729), UM-8D6 (https://www.abcam.com/cd147-antibody-8d6-ab194401.html), MEM-M6/6 (https://www.abcam.com/cd147-antibody-mem-m66-low-endotoxin-azide-free-ab119114.html) and AC-15 (https://www.abcam.com/beta-actin-antibody-ac-15-ab6276.html) had all been validated for use based on the supplier's recommendations (web links provided). Mouse anti-human CD62P was in routine use for assessing platelet activation and had been validated for use based on the supplier's recommendations (https://www.biolegend.com/en-us/products/pe-anti-human-cd62p-p-selectin-antibody-595).

Eukaryotic cell lines

Policy information about cell lines

Cell line source(s)

BEL-A2 (University of Bristol and NHS Blood and Transplant), HEK293T (European Collection of Authenticated Cell Cultures, catalogue no. 12022001) and Daudi cells (Lund University).

Authentication

BEL-A2 was extensively characterized, as published in Trakarnsanga et al, Nat Commun. 8: 14750, 2017. HEK293T has been authenticated for use based on the supplier's recommendations (https://www.phe-culturecollections.org.uk/products/celllines/generalcell/detail.jsp?refId=12022001&collection=ecacc_gc). The Daudi cell line is well known and the main character validated here is that it does not normally express MAM/EMP3.

Mycoplasma contamination

BEL-A2, HEK293T and DAUDI are mycoplasma negative, as tested at IBGRL, Bristol and Lund University.

Commonly misidentified lines
(See ICLAC register)

No commonly misidentified lines were used.

Human research participants

Policy information about studies involving human research participants

Population characteristics	The 10 individuals included in this study were women whom had been identified as MAM-negative following the identification of a mutually-compatible antibody in their respective plasma to a high-prevalence antigen that was, in all cases, identified as a consequence of pregnancy. While other MAM-negative individuals of both genders most certainly exist, it was the presence of anti-MAM that brought these samples to the attention of our laboratories.
Recruitment	The only individuals recruited for the purposes of this study were family members of P9 (F1, F2 and F3), following informed consent. For serological and genetic testing, fresh blood samples were obtained from F1, F2 and F3. For cell culture, control MAM-positive anonymized samples were waste products of blood donations; either buffy coats or whole blood that were used according to current regulations and local approval by the blood centre. Patient samples may have an inherent bias due to the way in which MAM-negative individuals are identified by antibody production due to previous pregnancy. Therefore, sex matched controls were selected were required.
Ethics oversight	All blood samples were procured and the study was conducted according to NHS Blood and Transplant (NHSBT) Research and Development governance requirements and ethical standards. Cell culture at Lund University was conducted with control donors following approval by the advisory board at the regional blood centre.

Note that full information on the approval of the study protocol must also be provided in the manuscript.

Flow Cytometry

Plots

Confirm that:

- [x] The axis labels state the marker and fluorochrome used (e.g. CD4-FITC).
- [x] The axis scales are clearly visible. Include numbers along axes only for bottom left plot of group (a 'group' is an analysis of identical markers).
- [x] All plots are contour plots with outliers or pseudocolor plots.
- [x] A numerical value for number of cells or percentage (with statistics) is provided.

Methodology

Sample preparation	Erythrocytes, BEL-A2 cells and cultured erythroid progenitor cells were labeled with the following antibodies: mouse monoclonal anti-EMP3 (Sigma-Aldrich) and anti-CD44 (BRIC222 and BRIC235; IBGRL Research Products). Isotype controls (BioRad) were tested in parallel to ascertain background fluorescence. For detection of MAM, human anti-MAM eluates were prepared by adsorption and elution from antigen-positive cells (Elu-Kit II; Gamma Biologicals). In all experiments, cells were incubated with primary antibody at room temperature for 30 min and washed once. Bound antibody was detected by adding fluorescein isothiocyanate or R-phycoerythrin anti–mouse globulins (Dako) or Alexa Fluor® 647-AffiniPure F(ab')2 fragment goat anti-human IgG (Jackson ImmunoResearch Europe) and incubated at 4°C for 30 min. Cells were washed once, and the fluorescence geometric mean was recorded for each test. Daudi cells in PBS with 1% BSA were tested with an anti-MAM eluate, or normal human IgG (as isotype control; R&D systems). Allophycocyanin (APC)-conjugated AffiniPure F(ab')2 fragment goat-anti-Human IgG (Jackson ImmunoResearch Europe) was used as secondary antibody. For platelet experiments an equal number of platelets from each sample was centrifuged at 800xg and washed twice with PBS. Each sample was stained with human anti-MAM eluate (1:4), human AB serum (1:4) or human IgG (0.2 µg/ml) for 30 min at room temperature. The platelets were washed with PBS containing 1% BSA and stained with mouse anti-human CD62P (PE conjugated, BD) (1:100) and goat anti-human secondary conjugated to APC (Jackson laboratory) (1:200) for 20 min at room temperature.
Instrument	Flow cytometry was performed on Navios instrument (Beckman Coulter), LSR Fortessa II cell cytometer, AriaIII cell sorter (Becton Dickinson) and FACSCanto II (Becton Dickinson).
Software	For flow cytometry acquisition: Navios System Software (Beckman Coultard) and for flow cytometry analysis: Kaluza v.1.2 and v.2.1 (Beckman Coultard); BD FACSDiva (Becton Dickinson), FlowJo v.10.4.2.
Cell population abundance	Mature red cell population was 100% pure, as determined by GPA expression. BEL-A2 and differentiating erythroid cells were acquired within this gate. 100% of a DAUDI cell line stably expressing GFP were acquired for analysis. A sample containing platelets obtained from a leucocyte-depleted plateletpheresis product prepared for clinical use was 100 % pure, as determined by platelet-specific antigen (CD61).

Gating strategy

FSC/SSC were acquired on a logarithmic scale. Relevant cell populations (red cells and BEL-A2) were identified using a red cell specific antibody (BRIC 256, IBGRL Research Products; GPA) as a marker of pure population and gated accordingly. Using isotype controls, negative populations were set to the first decade of the logarithmic fluorescent scale, and any positive populations were observed as the shift to the right beyond this point. For DAUDI Cells, FSC/SSC were acquired on a linear scale and further gating done using isotype controls, negative populations were set to the first decade of the logarithmic fluorescent scale, and any positive populations were observed as the shift to the right beyond this point. For Platelets, FSC/SSC were acquired on a logarithmic scale and further gating done using CD62P-negative and -positive to analyse MAM expression.
Gating strategy is provided in Supplementary Figure 10, in the Supplementary Information file.

☒ Tick this box to confirm that a figure exemplifying the gating strategy is provided in the Supplementary Information.
